# Caffeine intake exerts dual genome-wide effects on hippocampal metabolism and learning-dependent transcription

**DOI:** 10.1172/JCI149371

**Published:** 2022-06-15

**Authors:** Isabel Paiva, Lucrezia Cellai, Céline Meriaux, Lauranne Poncelet, Ouada Nebie, Jean-Michel Saliou, Anne-Sophie Lacoste, Anthony Papegaey, Hervé Drobecq, Stéphanie Le Gras, Marion Schneider, Enas M. Malik, Christa E. Müller, Emilie Faivre, Kevin Carvalho, Victoria Gomez-Murcia, Didier Vieau, Bryan Thiroux, Sabiha Eddarkaoui, Thibaud Lebouvier, Estelle Schueller, Laura Tzeplaeff, Iris Grgurina, Jonathan Seguin, Jonathan Stauber, Luisa V. Lopes, Luc Buée, Valérie Buée-Scherrer, Rodrigo A. Cunha, Rima Ait-Belkacem, Nicolas Sergeant, Jean-Sébastien Annicotte, Anne-Laurence Boutillier, David Blum

**Affiliations:** 1 University of Strasbourg, CNRS, UMR7364 — Laboratoire de Neurosciences Cognitives et Adaptatives (LNCA), Strasbourg, France.; 2 University of Lille, Inserm, CHU Lille, UMR-S1172 Lille Neuroscience & Cognition (LilNCog), Lille, France.; 3 Alzheimer and Tauopathies, LabEx DISTALZ, France.; 4 ImaBiotech SAS, Parc Eurasanté, Loos, France.; 5 University of Lille, CNRS, Inserm, CHU Lille, Institut Pasteur de Lille, UAR CNRS 2014 — US Inserm 41 — PLBS, Lille, France.; 6 Centre d’Infection et d’Immunité de Lille (CIIL) — INSERM U1019 — UMR 9017, Lille, France.; 7 University of Strasbourg, CNRS UMR7104, Inserm U1258 — GenomEast Platform — Institut de Génétique et de Biologie Moléculaire et Cellulaire (IGBMC), Illkirch, France.; 8 PharmaCenter Bonn, Pharmaceutical Institute, Pharmaceutical and Medicinal Chemistry, University of Bonn, Bonn, Germany.; 9 CHU Lille, Memory Clinic, Lille, France.; 10 Instituto de Medicina Molecular, Faculdade de Medicina de Lisboa, Universidade de Lisboa, Lisbon, Portugal.; 11 Center for Neuroscience and Cell Biology (CNC) and; 12 Faculty of Medicine, University of Coimbra, Coimbra, Portugal.; 13 University of Lille, INSERM, CNRS, CHU Lille, Institut Pasteur de Lille, Inserm U1283/CNRS UMR8199 — EGID, Lille, France.; 14 University of Lille, INSERM, CHU Lille, Institut Pasteur de Lille, U1167 — RID-AGE — Facteurs de risque et déterminants moléculaires des maladies liées au vieillissement, Lille, France.

**Keywords:** Neuroscience, Epigenetics, Memory, Pharmacology

## Abstract

Caffeine is the most widely consumed psychoactive substance in the world. Strikingly, the molecular pathways engaged by its regular consumption remain unclear. We herein addressed the mechanisms associated with habitual (chronic) caffeine consumption in the mouse hippocampus using untargeted orthogonal omics techniques. Our results revealed that chronic caffeine exerts concerted pleiotropic effects in the hippocampus at the epigenomic, proteomic, and metabolomic levels. Caffeine lowered metabolism-related processes (e.g., at the level of metabolomics and gene expression) in bulk tissue, while it induced neuron-specific epigenetic changes at synaptic transmission/plasticity-related genes and increased experience-driven transcriptional activity. Altogether, these findings suggest that regular caffeine intake improves the signal-to-noise ratio during information encoding, in part through fine-tuning of metabolic genes, while boosting the salience of information processing during learning in neuronal circuits.

## Graphical abstract

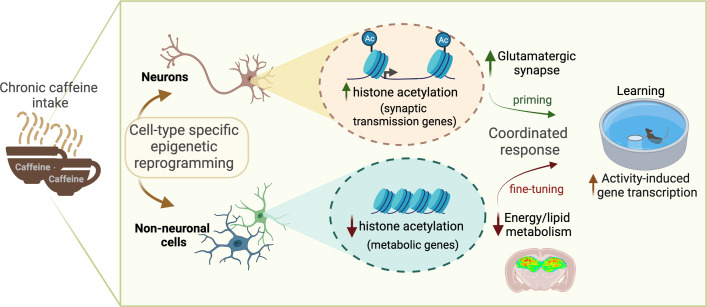

## Introduction

Caffeine is the most widely consumed psychoactive substance in the world (by about 80% of the population) via dietary intake from coffee, tea, and soda beverages. Its popularity derives from the ability to enhance well-being and some CNS-related functions such as attention and alertness (1). Large epidemiological studies point to an inverse association between coffee/caffeine consumption and all-cause mortality (2–4). In general, the effect of caffeine on human health follows an inverted bell-shaped dose-response curve, with benefits observable at doses of 200-400 mg/d that can be recapitulated by 0.3 g/L p.o. in rodents.

Compelling epidemiological and experimental evidence supports that habitual/chronic caffeine consumption normalizes synaptic plasticity and reduces cognitive decline in altered allostatic situations such as aging, Alzheimer’s disease, and other neuropsychiatric conditions (5–7). A more limited number of studies, however, also support that, independent of its ability to favor arousal and attention, caffeine may exhibit cognition-enhancing properties. After being rewarded with caffeine, honeybees are able to remember a previously learned floral scent (8). Also, acute caffeine administration in rats can enhance memory test performance (9, 10). In humans, caffeine intake immediately following learning improves discrimination performance 24 hours later (11). These results are in line with observations supporting the ability of caffeine to modulate hippocampal/cortical excitability in homeostatic conditions. Indeed, caffeine treatment in hippocampal slices enhances basal synaptic transmission (12–14), and in the rodent hippocampus (12, 15, 16) modulates long-term potentiation (LTP) and sharp wave–ripple complexes, which are proposed to underlie memory consolidation (17). Caffeine also controls neuronal excitability and LTP-like effects in the human cortex (18, 19). Most of these studies, however, rely on acute administration, with limited relevance for habitual/chronic consumption.

Despite caffeine’s popularity, brain molecular changes associated with its chronic intake remain ill defined. Caffeine is known to essentially interfere with the adenosinergic system, where it acts as an antagonist (20). However, adaptive downstream pathways engaged by habitual/chronic caffeine consumption have been largely overlooked. In the present study, we used a combination of unbiased orthogonal omics techniques to analyze the epigenome, transcriptome, proteome, and metabolome of the mouse hippocampus in order to uncover the molecular pathways affected by chronic caffeine consumption in neuronal processing during learning.

## Results

### Mouse monitoring and caffeine concentrations.

In our experimental conditions, neither mortality nor signs of suffering were encountered in caffeine-treated animals. Average consumption of 0.3 g/L caffeinated water was 4.83 ± 0.15 mL/mouse/d, resulting in brain caffeine concentrations of 3.6 ± 1.1 μM, corresponding to moderate intake in humans (20). Caffeine metabolites (paraxanthine, theobromine, and theophylline) were also detected in the brain of treated mice, with respective concentrations of 1.9 ± 0.4 μM, 1.8 ± 0.3 μM, and 0.10 ± 0.03 μM (*n* = 5).

### Chronic caffeine consumption decreases histone acetylation of metabolism-related genes in the hippocampus.

**Figure 1:**
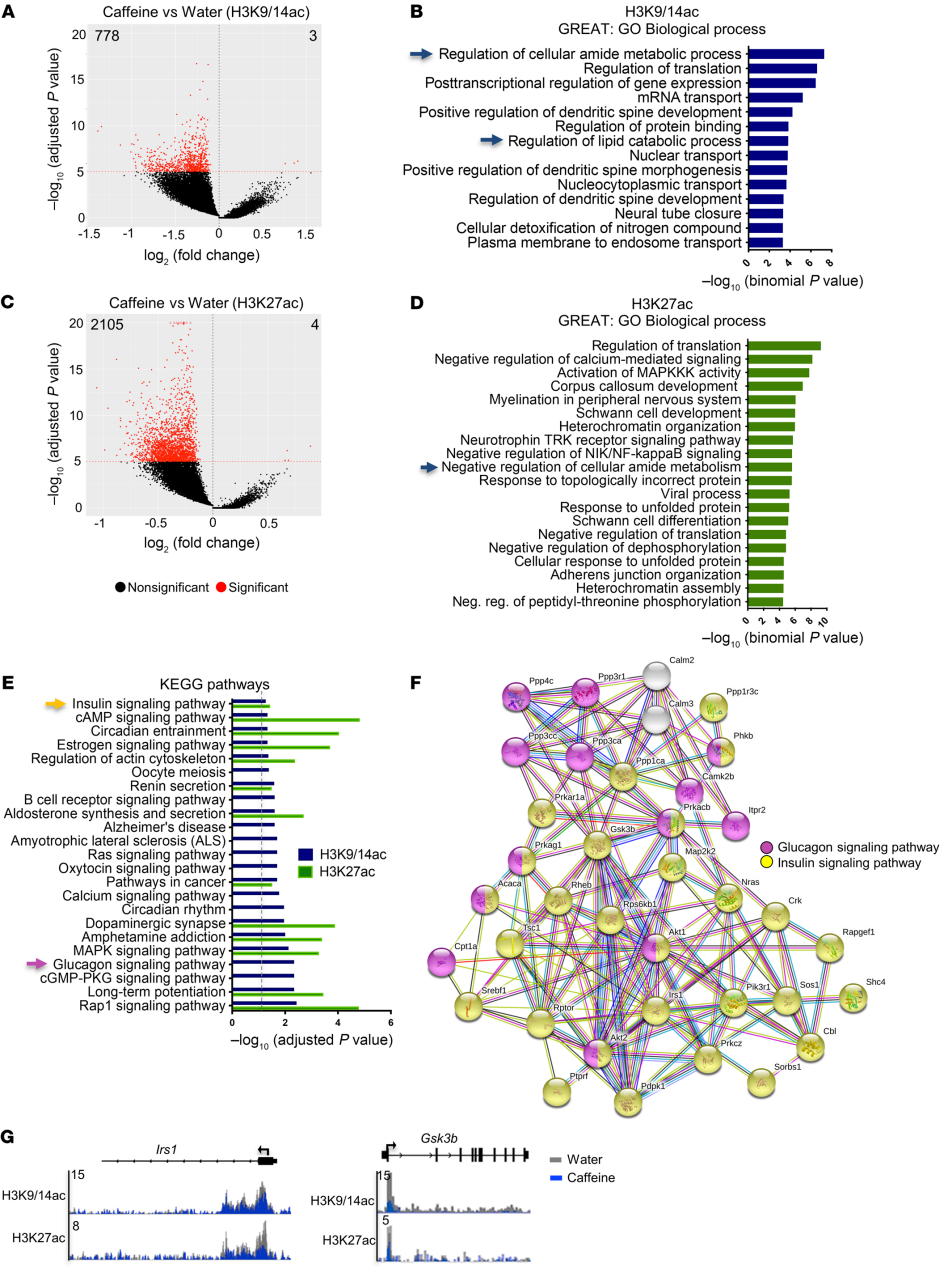
Hippocampal epigenomic alterations associated with chronic caffeine consumption. (**A**) Volcano plot showing the differentially enriched genomic regions of H3K9/14ac (ChIP-Seq) upon chronic caffeine treatment (778 decreased and 3 increased peaks, FDR < 1 × 10^–5^). (**B**) GREAT analysis showing the most-enriched biological processes associated with the H3K9/14ac-decreased peaks in caffeine-treated mice. Blue arrows indicate metabolic process– and translation-related terms. (**C**) Volcano plot representing the differentially regulated regions of H3K27ac upon chronic caffeine treatment (2105 decreased and 4 increased peaks, with FDR < 1 × 10^–5^). (**D**) GREAT analysis representing the most common biological processes associated with the H3K27ac-decreased peaks in the caffeine group. Regulation of metabolic processes is indicated by the blue arrow. (**E**) KEGG pathway analyses of depleted regions of both histone marks. Dashed gray line indicates adjusted *P* < 0.05. Yellow and pink arrows correspond to highlighted pathways in **F**. (**F**) Functional protein-protein network analysis (STRING) representation of insulin- and glucagon-related genes found to be decreased in both histone acetylation marks. (**G**) A representation of genomic regions using Integrative Genomics Viewer (IGV) of the metabolic genes *Irs1* and *Gsk3b*, showing significant (FDR<10-5) decreases in H3K27ac and H3K9/14ac after caffeine treatment. Two biological replicates per histone mark were used for ChIP-Seq experiments.

We hypothesized that chronic caffeine consumption could affect the mouse hippocampal epigenome. As caffeine is a psychostimulant, we focused on 2 chromatin marks associated with active chromatin and specific transcriptional states. Histone H3 acetylation at lysine 27 (H3K27ac) is preferentially enriched at active enhancers (21), also forming large clusters of H3K27ac-enriched enhancers known as super-enhancers on highly transcribed genes that are cell- or tissue-specific (22, 23). The histone H3 lysines K9 and K14 (H3K9/K14ac), on which acetylation co-occurs at many gene-regulatory elements, allows differentiation of active from inactive enhancers and thus represents a dynamic mark accounting for stimulus-dependent activation (24). Locus-specific acetylation was evaluated by ChIP-Seq experiments in the dorsal hippocampus of control- (water-) and caffeine-treated mice. A total of 2 biological replicates were used, and principal component analysis (PCA) plots of the 2 histone marks were generated (Supplemental Figure 1, A and B; supplemental material available online with this article; https://doi.org/10.1172/JCI149371DS1). Chronic caffeine intake significantly decreased acetylation of both histone marks at many genomic loci. H3K9/14ac was depleted in 778 genomic regions (768 genes), while only 3 were identified as significantly enriched in caffeine-treated animals (FDR < 1 × 10^–5^; Figure 1A and Supplemental Table 1). Gene ontology analysis using the Genomic Regions Enrichment of Annotations Tool (GREAT) revealed that these acetylation-depleted regions were associated with genes involved in regulation of metabolic processes (amide, lipid); in mRNA transport; and in regulation of translation and dendritic spine morphogenesis and development (Figure 1B). A more robust effect was observed for H3K27ac, whose peaks were found to be decreased in 2105 (1766 genes) and increased in only 4 genomic regions in caffeine- versus control-treated mice (FDR < 1 × 10^–5^; Figure 1C and Supplemental Table 2). Metabolism-related pathways, such as lipid catabolic or amide metabolic processes, were among the decreased peaks of both histone marks (Figure 1, B and D). Additionally, H3K27ac-depleted regions were significantly associated with myelin-related processes, MAPK activity, negative regulation of calcium-mediated signaling pathways, and heterochromatin organization (Figure 1D). We also performed Kyoto Encyclopedia of Genes and Genomes (KEGG) pathway analyses and identified many processes, some of which related to cAMP, MAP kinase, and Rap1 signaling pathways and circadian entrainment for both H3K9/14ac- and H3K27ac-depleted regions (Figure 1E). Of note, the KEGG pathway database indicated metabolism-related pathways, such as “insulin signaling,” for genes depleted in acetylation of both histone marks; and “glucagon signaling pathway” for those associated with H3K9/14ac-depleted regions (Figure 1E). Those genes associated with insulin and glucagon signaling pathways were represented by protein-protein interaction network analysis (STRING; https://string-db.org/), showing strong interconnectivity (Figure 1F, yellow and pink dots, respectively). A representation of genomic regions using Integrative Genomics Viewer (IGV) is shown for the insulin receptor substrate 1 (*Irs1*) gene, which is required for insulin signaling and related spine maturation and synaptic plasticity (25); and the glycogen synthase kinase 3β (*Gsk3b*) gene (Figure 1G). Both genes present significant acetylation depletion of H3K9K14ac and H3K27ac in the caffeine- versus control-treated group (respectively, *Irs1*, H3K9/14ac, FDR = 7.75 × 10^–5^ and H3K27ac, FDR = 1.82 × 10^–12^; *Gsk3b*, H3K9/14ac, FDR = 2.58 × 10^–11^ and H3K27ac, FDR = 4.83 × 10^–5^). Other regions, such as those associated with the *Dusp3*, *Psme3*, and *Mlh3* genes, did not exhibit such histone acetylation changes upon caffeine treatment, attesting to the selectivity of the caffeine effect for both histone marks (Supplemental Figure 1C). In addition, integrated pathway analysis (IPA) applied to ChIP-Seq data common to both marks confirmed that canonical metabolic pathways, such as insulin and IGF-1 signaling, were affected by histone acetylation depletion upon caffeine treatment (Supplemental Table 3). Potential contributors to the effects of caffeine on the epigenome were further assessed using the “upstream regulator analysis” function of IPA (Supplemental Table 4). Among the acetylation-depleted genes, we identified the transcription factor 7-like 2 (*TCF7L2*) as the most significantly affected upstream regulator for both marks upon caffeine consumption. Furthermore, ADORA2A (A2AR) was identified as another upstream regulator in the epigenomic data, in striking accordance with the primary ability of caffeine to antagonize adenosine receptors (20). Altogether, these data show that in the bulk hippocampus, chronic caffeine treatment induces an overall deacetylation of 2 active transcription marks, H3K27ac and H3K9/14ac, on genes related to translation and lipid- and glucose/insulin-related metabolism.

To assess whether this histone acetylation depletion exerts an effect on gene transcription, we performed RNA-Seq of both water- and caffeine-treated mice. Although differential expression analysis revealed no statistically significant differences in gene expression between groups in individual H3K27ac-depleted genes (Supplemental Figure 2A), a significant decrease in overall expression (*z* score) of the 1776 H3K27ac-depleted genes was found in the caffeine group. A *z* score analysis using the same number of randomly selected genes revealed no changes in expression between groups (Supplemental Figure 2B), thus confirming specificity. Furthermore, we also checked by real-time quantitative analysis (qPCR; *n* = 5–6/group) expression levels of several genes chosen among those most depleted in H3K27ac and observed decreased expression following chronic caffeine treatment (Supplemental Figure 2D, red bars). Importantly, we found that some of these genes, such as PBX homeobox 1 (*Pbx1*), NAD kinase 2 (*Nadk2*), and spindle and centriole associated protein 1 (*Spice1*), displayed decreased expression not only upon chronic (2-week) but also following an acute (24-hour) caffeine treatment (Supplemental Figure 2D, green bars). However, the cytochrome P450 family 51 subfamily A member 1 (*Cyp51*) gene, which plays a central role in cholesterol and lipid metabolism, showed decreased expression solely upon chronic caffeine treatment. Moreover, a persistent effect of caffeine on gene expression was observed for the *Pbx1* and *Nadk2* genes, as their expression remained decreased even after a 2-week caffeine withdrawal following chronic administration (Supplemental Figure 2D, blue bars).

### Effect of chronic caffeine consumption on the hippocampal metabolome.

**Figure 2:**
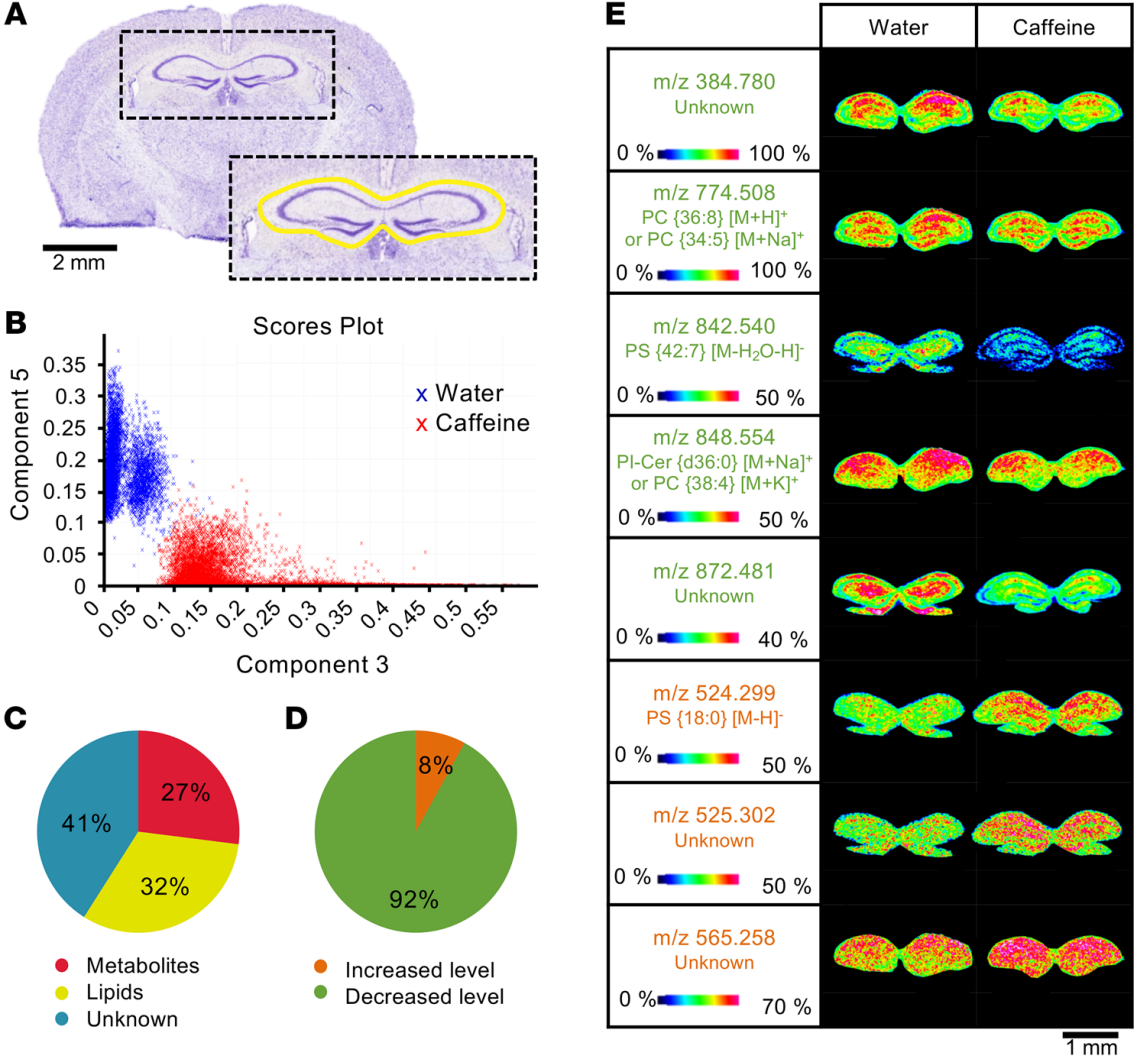
Hippocampal metabolomic changes induced by chronic caffeine consumption. (**A**) Unsupervised PCA performed in the hippocampal region of interest (indicated in yellow) on Nissl-stained brain tissue sections. Scale bar: 2 mm. (**B**) Scores from the unsupervised PCA in the hippocampus of water- and caffeine-treated mice are presented in a plot where the differences between the molecular signatures of the 2 experimental groups clearly emerge. Pie charts showing the distribution of the different classes of molecules (**C**) and the changes in their levels (**D**) based on *m/z* measured in positive or negative ionization modes, with a significant quantitative difference after Student’s *t* test analysis in the hippocampus of caffeine- compared with water-treated animals (*n* = 6/group). (**E**) MS images obtained at a spatial resolution of 35 μm for *m/z* resenting a decreased (green) or increased (orange) density in the hippocampus of caffeine- compared with water-treated mice. The color scale shows the intensity of the *m/z* of interest. Cer, ceramide; PC, phosphatidylcholine; PI, phosphatidylinositol; PS, phosphatidylserine. Scale bar: 1 mm.

Considering that caffeine decreased histone acetylation of metabolism-related genes, we further assessed the effect of the decreased histone acetylation on the hippocampal metabolome. To do so, we visualized tissue spatial distribution of molecules by MALDI mass spectrometry (MS) imaging (MALDI-MSI) analysis, acquired from the dorsal hippocampus (bregma –1.7 mm; Figure 2A) of water- and caffeine-treated mice (*n* = 6/group). PCA analysis was then performed on the recorded MS images from both mouse groups (water and caffeine treated) in order to highlight differences in their hippocampal molecular distribution profiles (Figure 2B). This revealed lipidomic and metabolomic signatures related to chronic caffeine intake, resulting in 2 distinctly separated clusters. The identification of metabolites and lipids was based on measurement of their mass-to-charge ratios (*m/z*) and subsequent comparison with different data banks. In total, 59% of the metabolome was assigned to the biochemical classes of metabolites (27%) and lipids (32%; Figure 2C). The *m/z* value of the remaining 41% did not allow for unambiguous assignment to a specific biochemical class. Ultimately, statistical analysis of the molecular data sets revealed that chronic caffeine consumption induced a major decrease in metabolites and lipid levels (92% decreased vs. 8% increased; Figure 2D). The identified metabolites between the water and caffeine groups (*P* < 0.05), detected in positive and negative ionization mode, are listed in Supplemental Table 5. Representative molecular images showing different levels and distribution of some detected metabolites and lipids from hippocampi of water and caffeine-treated mice are shown in Figure 2E.

### Proteomic hippocampal signature associated with chronic caffeine consumption.

**Figure 3:**
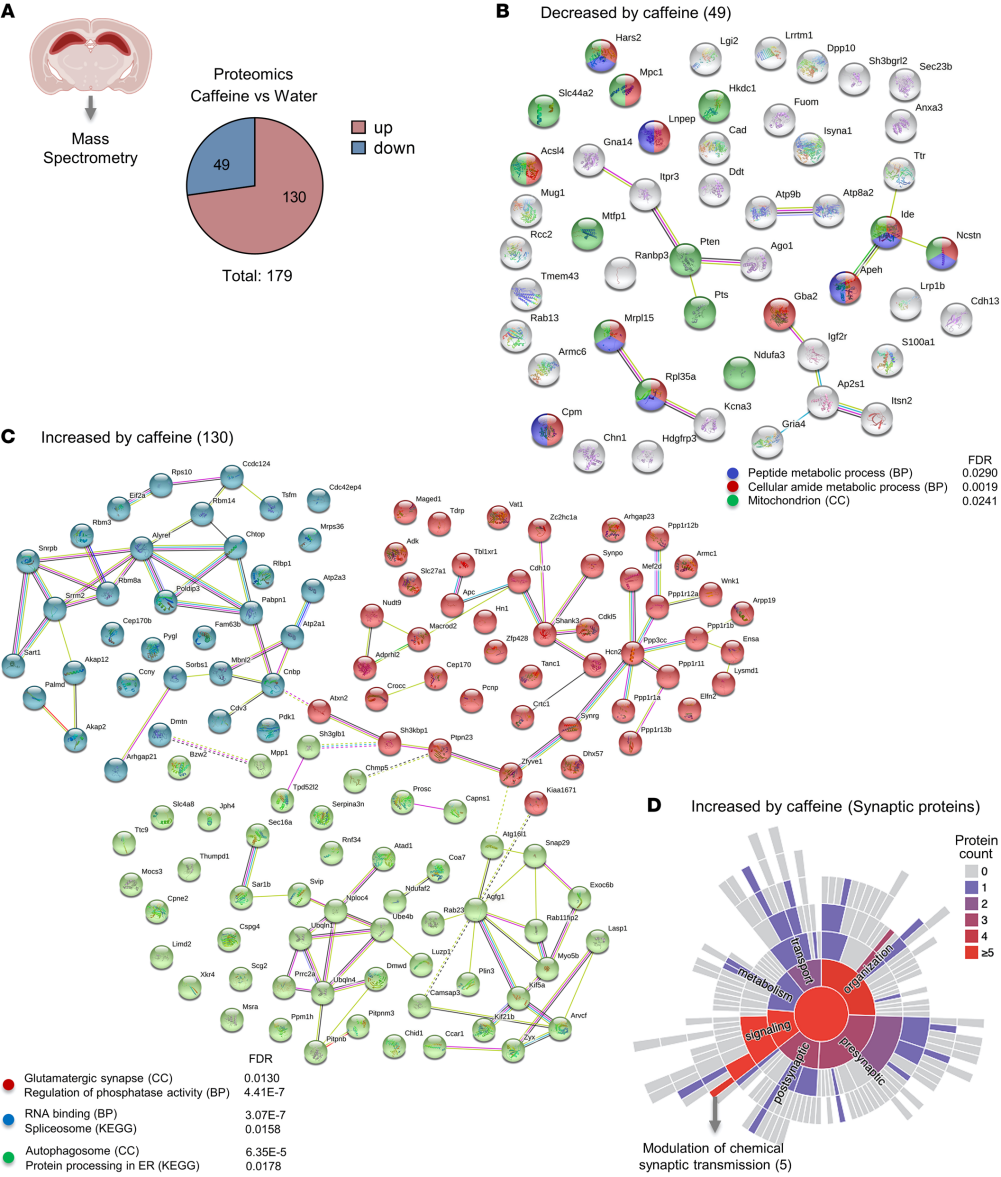
Alteration of hippocampal proteomics induced by chronic caffeine consumption. (**A**) Pie chart indicating proteins altered in the hippocampus of water- and caffeine-treated mice determined by MS analysis (*n* = 3/group). In total, 179 proteins were altered, of which 49 were decreased and 130 increased by chronic caffeine. (**B**) STRING network analysis of the 49 decreased proteins in the caffeine condition showing that they were associated with metabolism- and mitochondrion-related terms. (**C**) STRING network analysis of the 130 increased proteins by chronic caffeine revealing 3 major clusters (k-means). The cluster in red shows significance for glutamatergic synapse–related terms; the blue cluster represents proteins associated with RNA binding; the green, autophagosome-related pathways. BP, biological processes; CC, cellular component. (**D**) The SynGO ontologies and annotations (26) tool revealed that most of the synaptic proteins among the proteins increased by chronic caffeine are associated with synaptic signaling and modulation of chemical synaptic transmission. Warmer colors represent the predominance of proteins associated with the respective pathway.

To gain insights into the potential effect of chronic caffeine intake at the protein level, we performed MS proteomic analysis of the bulk dorsal hippocampus of water- (control-) and chronic caffeine–treated mice (*n* = 3/group). Caffeine induced alterations of 179 proteins, of which 49 showed decreased and 130 increased expression levels (Figure 3A and Supplemental Table 6). In line with the 2 previous data sets (epigenomics and metabolomics), gene ontology and protein network analysis revealed that decreased protein levels were again associated with peptide and cellular amide metabolic processes, as well as with mitochondria — with a reduction in NADH:ubiquinone oxidoreductase subunit A3 (NDUFA3), which is involved in mitochondrial respiratory chain complex I assembly; in mitochondrial pyruvate carrier 1 (MPC1), responsible for transporting pyruvate into mitochondria; and in long-chain fatty acid CoA ligase 4 (ACSL4), involved in lipid metabolism (Figure 3B). Together, these 3 approaches suggest a robust change in metabolic processes induced by chronic caffeine intake in bulk hippocampal tissue. Decreases in levels of 35 of the 49 proteins with caffeine treatment, including insulin-degrading enzyme (IDE) and NDUFA3, were reversed by caffeine withdrawal. Only 14 proteins, including IGF2R, remained decreased following caffeine withdrawal (Supplemental Table 6).

Gene ontology analysis of the increased proteins revealed 3 main protein clusters: one related to RNA binding and the spliceosome, a second linked to the autophagosome and protein processing in the endoplasmic reticulum, and a third associated with glutamatergic synapse and phosphatase activity (Figure 3C). Considering that caffeine induced expression of some synaptic proteins — and controls glutamatergic synaptic transmission (e.g., ref. 19) — we further assessed their predicted role in the synaptic compartment using SynGO ontologies and annotations (26). We observed that most of the synaptic proteins annotated were related to synaptic organization and signaling (Figure 3D) and, more particularly, to chemical synaptic transmission, such as SH3 and multiple ankyrin repeat domains 3 (SHANK3), which encodes critical scaffolding proteins for glutamatergic neurotransmission in postsynaptic densities (27); synaptopodin (SYNPO), a part of the actin cytoskeleton of postsynaptic densities (28); and CREB–regulated transcription coactivator 1 (CRTC1), involved in hippocampal plasticity and memory (29). Overall, proteomic analysis revealed a decrease in levels of metabolism-related proteins, concomitant with an increase in neuron/synapse-associated proteins. Interestingly, increases in levels of 57 of the 130 proteins with chronic caffeine intake, including SHANK3 and SYNPO, were reversed 2 weeks after caffeine withdrawal, while the increased levels of the other 73 proteins persisted. The latter included the ATPase family AAA domain containing 1 (ATAD1) protein, which controls synaptic plasticity by regulating the release of neurotransmitter receptors from postsynaptic scaffolds (ref. 30 and Supplemental Table 6).

### Neuron-specific H3K27ac in synaptic transmission–related genes is increased by chronic caffeine consumption.

**Figure 4:**
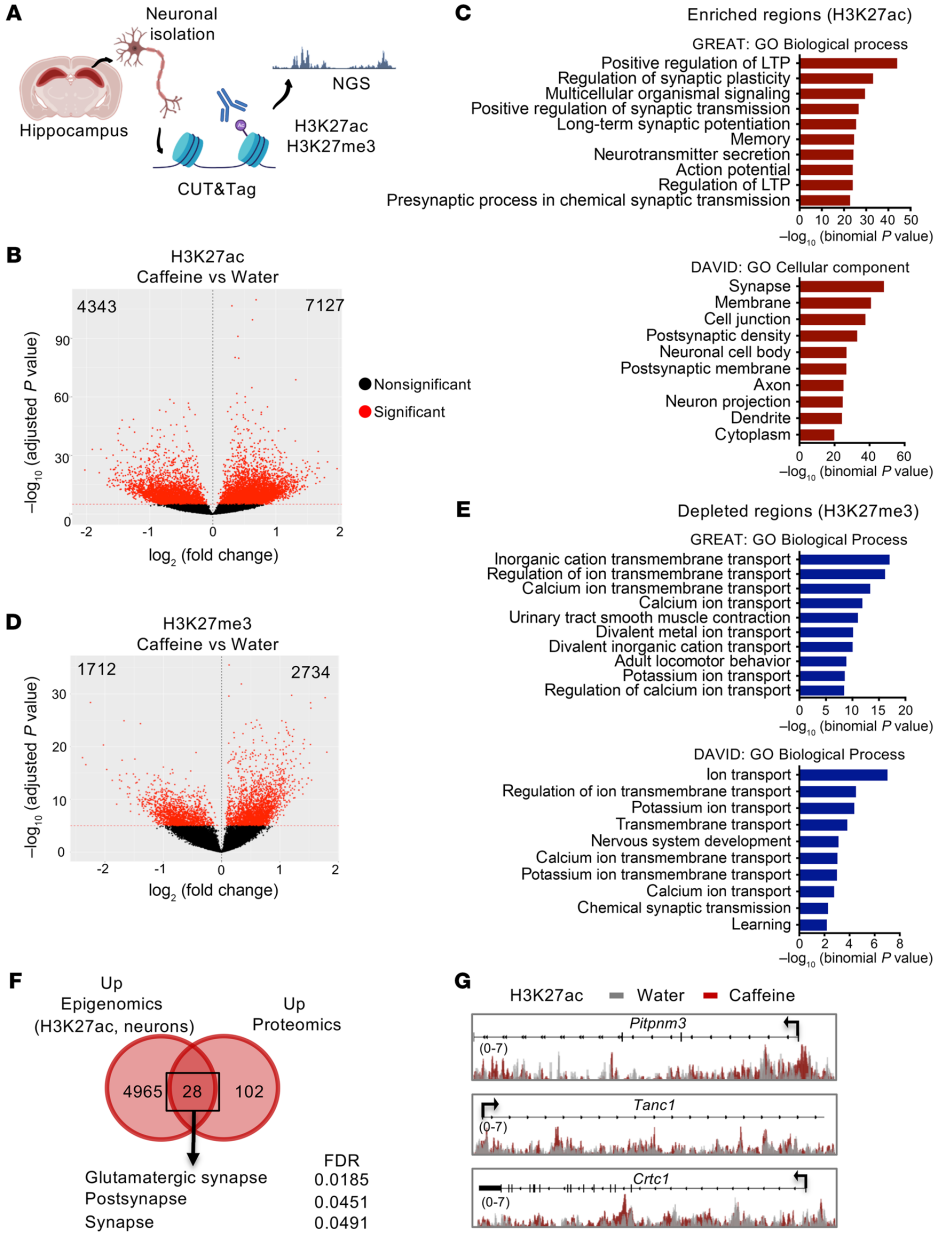
Neuron-specific H3K27ac and H3K27me3 changes induced by chronic caffeine consumption. (**A**) Schematic of the experimental design used to assess the active (H3K27ac) and repressive (H3K27me3) histone marks by the CUT&Tag technique in a hippocampal neuron-enriched population. NGS, next-generation sequencing. (**B**) Volcano plot representing H3K27ac differentially regulated regions (4343 depleted and 7127 enriched, FDR < 1 × 10^–5^). (**C**) Top: GREAT analysis showing the most-enriched biological processes associated with the H3K27ac-enriched peaks in caffeine-treated mice, primarily related to synaptic transmission. Bottom: DAVID Gene Ontology analysis revealing the most significant cellular components associated with H3K27ac-enriched regions. (**D**) Volcano plot showing H3K27me3 differentially regulated regions between the water- (control) and caffeine-treated mouse hippocampus (1712 depleted and 2734 enriched, FDR < 1 × 10^–5^). (**E**) Top: GREAT analysis showing that depleted regions are mostly associated with ion transport processes. Bottom: DAVID Gene Ontology analysis indicating the most significant biological processes associated with H3K27ac-enriched genes in neurons. (**F**) Venn diagram showing that 28 proteins were increased by caffeine and enriched in H3K27ac at their coding genes. These proteins are mostly associated with glutamatergic synapse (STRING analysis). (**G**) Representation (using IGV) of H3K27ac-enrichement of genomic regions at 3 genes in neurons from mice treated with water or chronic caffeine. Two biological replicates per histone mark were used for CUT&Tag experiments.

Our epigenomic data from experiments performed in bulk tissue revealed a robust decrease in histone acetylation (H3K27ac and H3K9/14ac; Figure 1). However, the increased synaptic protein levels observed following MS proteomics in caffeine-treated mice supported a presumably neuron-autonomous effect of caffeine and underscored the importance of conducting cell type–specific experiments to better understand caffeine-induced adaptive alterations of neuronal/synaptic transmission. Thus, to assess cell-specific effects, we analyzed the epigenome of neuron-enriched populations derived from water- and caffeine-treated mouse dorsal hippocampi. Instead of ChIP-Seq, we used a novel enzyme-tethering strategy, the Cleavage Under Targets and Tagmentation (CUT&Tag) approach, which allows profiling of a lower number of cells at a higher resolution (31). We first validated the CUT&Tag-Seq approach by analyzing H3K27ac signatures in a hippocampal cell suspension (“all cells”) and compared the results with ChIP-Seq results obtained in bulk tissue. In all cells, CUT&Tag-Seq analysis revealed a strong depletion of H3K27ac in caffeine-treated mice as observed by ChIP-Seq (Supplemental Figure 3A). IPA analysis of the depleted H3K27ac genes revealed common upstream regulators (TCF7L2, MAP kinase–interacting serine/threonine kinase 1 [MKNK1], neurofascin [NFASC]) with these 2 different experimental designs (Supplemental Figure 3B). Additionally, a high degree of overlap was found for the KEGG pathways of the common acetylation-depleted genes obtained with CUT&Tag and ChIP-Seq analyses (Supplemental Figure 3C). We proceeded with the neuronal epigenetic study by CUT&Tag-Seq using H3K27ac, as this mark displayed the stronger caffeine-associated alterations in bulk ChIP experiments. To further support our results, we investigated a repressive version of this mark, trimethylation of H3 histone at lysine 27 (H3K27me3) (Figure 4A). We first confirmed that neuronal genes were enriched in H3K27ac and depleted in H3K27me3 in our neuron-enriched cellular fraction, while the opposite was seen for a set of glia-associated genes (Supplemental Figure 4A). In sharp contrast with data obtained from bulk hippocampal tissue, differential analyses of caffeine-treated and control mice revealed a preponderance of H3K27ac-enriched regions in neurons obtained from caffeine-treated mice (7127, FDR < 10 × 10^–6^) as compared with the number of depleted regions (4343, FDR < 10 × 10^–6^) (Figure 4B and Supplemental Table 8). An H3K27ac PCA plot indicated a separation between the 2 groups, with a PC1 of 58% (Supplemental Figure 4B). While depleted regions were found mostly in genes associated with the immune response (Supplemental Figure 5A), enriched regions were strongly related to the synaptic compartment involved in the regulation of synaptic plasticity, action potential, LTP, and memory (see DAVID: GO Cellular component; and GREAT: GO Biological process) (Figure 4C). The repressive mark H3K27me3 also displayed a higher number of enriched (2734, FDR < 10 × 10^–6^) than depleted regions (1712, FDR < 10 × 10^–6^) (Figure 4D and Supplemental Table 8). An H3K27me3 PCA plot indicated a separation between the 2 groups, with a PC1 of 53% (Supplemental Figure 4C). Strikingly, H3K27me3-depleted region–associated genes in neurons were primarily linked to ion transport processes such as calcium and potassium transport, as well as chemical synaptic transmission and learning (Figure 4E), while enriched regions were associated with transcription and histone deacetylase binding–related processes (Supplemental Figure 5B). Analysis of the intersecting regions depleted in acetylation and increased in methylation showed 282 regions that corresponded to genes linked to the transcription machinery (Supplemental Figure 5, C and D, blue). The converse analysis, showing overlapping regions enriched in acetylation and depleted in methylation, represented 352 regions that were linked to ion transport functions (Supplemental Figure 5, C and D, red). This suggests that pathways linked to synaptic transmission, learning, and regulation of membrane potential are coregulated in neurons by chronic caffeine treatment, leading to H3K27 acetylation enrichment and trimethylation depletion, also clearly visualized by cluster profiling representation (Supplemental Figure 5E). Finally, integration between neuron-specific epigenomic data and proteomic data showed that 28 of the 130 proteins increased by caffeine exhibited significant H3K27ac enrichment at their coding sequence, mostly related to synapses, particularly to glutamatergic synapses (Figure 4F). These 28 genes/proteins include the calcium binding protein membrane-associated phosphatidylinositol transfer protein 3 (*Pitpnm3*); as well as tetratricopeptide repeat, ankyrin repeat and coiled-coil containing 1 (*Tanc1*), which is a PSD-95–interacting protein regulating dendritic spines at excitatory synapses (32). Among these 28 genes/proteins, we also identified CREB-regulated transcription coactivator 1 (*Crtc1*), which is required for efficient induction of CREB target genes to engage activity-dependent transcription during neuronal activity (ref. 33 and Figure 4G). Overall, these findings suggest that chronic caffeine intake exerts cell-autonomous positive epigenetic modulation of the synaptic transmission and plasticity processes in hippocampal neurons.

### Chronic caffeine consumption enhances learning-dependent hippocampal transcription.

**Figure 5:**
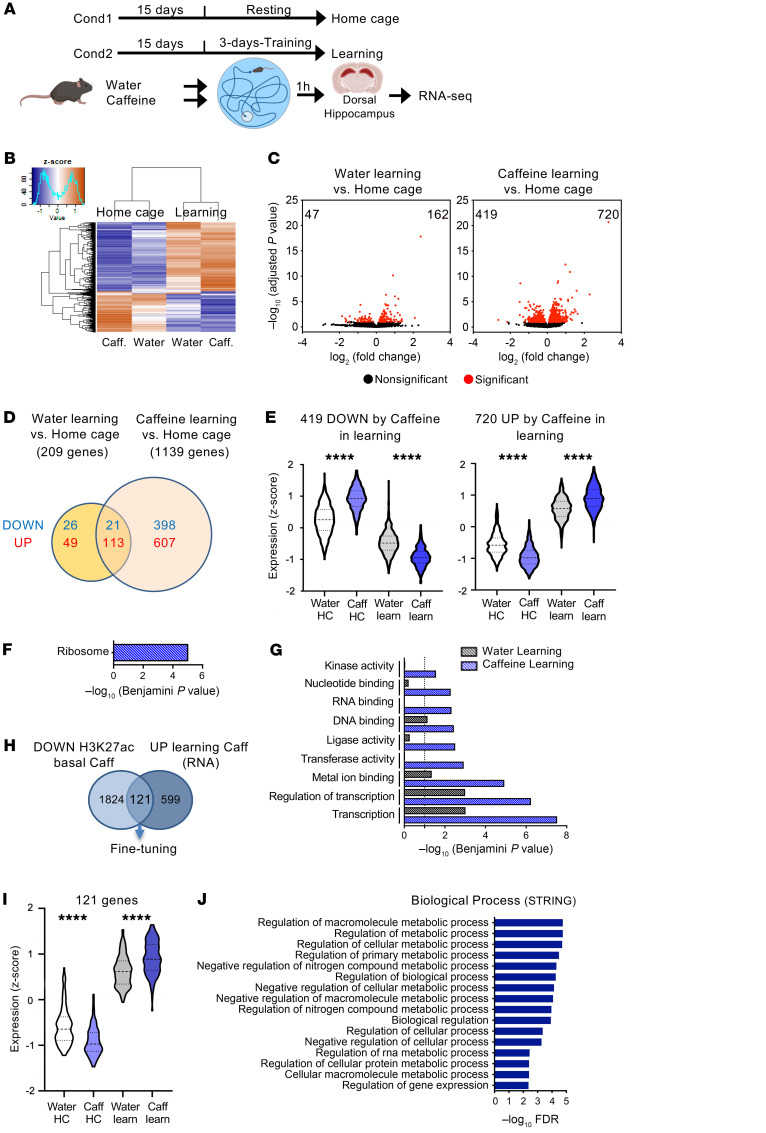
Hippocampal transcriptomic alterations induced by chronic caffeine consumption in learning conditions. (**A**) Experimental procedure for the RNA-Seq experiments in the home cage and learning groups. After chronic consumption of caffeine (or water as a control), mice were subjected to 3 days of training in the MWM, and the dorsal hippocampus was dissected 1 hour after the last trial for RNA-Seq. Cond, condition. (**B**) Heatmap representation of RNA-Seq results (*z* score) in the 4 groups. A total of 4 biological replicates were used per group. Color coding was performed according to the *z* score of the normalized read counts divided by gene length. (**C**) Left: Volcano plots show the differentially expressed hippocampal genes (adjusted *P* < 0.1) between water-treated control learning and control home cage groups. Right: Volcano plot showing differentially expressed genes in caffeine-treated learning and home cage mice (adjusted *P* < 0.1). (**D**) Venn diagram showing the transcriptome changes induced by learning in water- and caffeine-treated animals (adjusted *P* < 0.1). (**E**) Violin plots representing expression values (*z* score) of the 419 genes downregulated and the 720 upregulated by caffeine (Caff) in learning (learn), showing opposite trends among the home cage (HC) groups. (**F**) KEGG pathway analysis showing that most of the genes downregulated by caffeine upon learning are associated with ribosome. (**G**) Functional annotation performed with DAVID, and significance for the effect of learning in water- and caffeine-treated animals. (**H**) Venn diagram revealing 121 genes depleted in H3K27ac (bulk hippocampus ChIP-Seq; DOWN) and upregulated (RNA; UP) by learning in caffeine-treated mice. (**I**) Violin plots of the expression values (*z* score) of the 121 genes. (**J**) Gene ontology analysis performed with STRING of the 121 genes, showing a strong association with metabolism-related biological processes (top 16 by FDR). Statistical significance in **E** and **I** was calculated by 1-way ANOVA followed by Bonferroni’s multiple-comparison post hoc test;****P < 0.0001.

Finally, we aimed at addressing whether chronic caffeine consumption had an effect on transcriptional regulation induced by learning processes. Two experimental conditions were assessed: “home cage,” consisting of resting mice; and “learning,” consisting of 3 days of training for spatial memory using the Morris water maze (MWM), a hippocampus-dependent task (Figure 5A). The learning groups showed better acquisition of the hidden platform position on the third day of training (d3), revealed by the decreased distance traveled during the last day (Supplemental Figure 6). Caffeine-treated animals spent significantly less time in the thigmotaxic zone than water-treated mice. The mean swim speed per day was similar in both learning groups. RNA-Seq analyses were then performed in the dorsal hippocampus in both the home cage and learning conditions (3 days of training + 1 hour: ref. 34) (Figure 5B; *n* = 4/group). Interestingly, when the response to training (learning vs. home cage conditions) was evaluated, differences emerged between the water- and caffeine-treated groups. While expression of 209 genes was significantly modified by learning in the water group (47 down- and 162 upregulated), in the caffeine-treated mice expression of genes was altered about 5 times more in response to learning, i.e., 1139 (419 down- and 720 upregulated) (Figure 5, C and D, and Supplemental Table 9). While in the resting state no genes had been found to be significantly modified by chronic caffeine treatment (Supplemental Figure 2A), the group of 419 genes that were significantly downregulated by learning had increased home cage– and decreased learning–mediated expression levels upon caffeine treatment (*z* scores), thus showing a greater amplitude of expression (Figure 5E). Likewise, higher amplitude of expression was observed for genes upregulated upon learning (720 upregulated), as they presented decreased expression levels in resting mice and increased expression levels in response to training (Figure 5E). The 419 genes downregulated by caffeine plus training were significantly associated with the “ribosome” KEGG pathway (Figure 5F). As we previously found that chronic caffeine treatment pointed to the common GO Biological Process term “translation” (ChIP-Seq data, Figure 1, B and D) and the common potential upstream regulator MKNK1 (Supplemental Tables 4 and 7), these RNA-Seq data indicate that chronic caffeine treatment may have a functional effect on the general translation processes in resting mice that can be amplified when the system is activated. The same reasoning holds true for genes that were induced upon chronic caffeine treatment and training: genes induced by training in water-treated animals were associated with transcriptional processes (Figure 5G) similarly to the caffeine-treated group (Figure 5H); however, caffeine treatment led to an increase in significance (Benjamini *P* value) associated with transcription-related pathways in response to learning compared with water treatment (Figure 5G). Accordingly, the immediate early gene *Fosb* and the *Xbp1* gene, which is known to play an important role in memory formation (35), were significantly more activated by the learning process under caffeine treatment (Supplemental Figure 7A). Caffeine treatment also promoted activation of other pathways related to metal ion binding or transferase/ligase/kinase activities (Figure 5G). Among the 607 genes specifically upregulated in caffeine-treated animals under learning conditions, we identified *Vegfa*, an important modulator of hippocampal neurogenesis and cognition (36); and *Acss1*, an acetyl-CoA synthetase–coding gene whose related family member *Acss2* regulates histone acetylation and hippocampus-dependent memory (ref. 37 and Supplemental Figure 7B). Further integration of the 720 genes upregulated by learning in caffeine-treated animals revealed that 121 of them were already deacetylated (H3K27ac ChIP-Seq) in resting conditions (Figure 5H), consistent with their decreased expression (*z* score) levels in home cage caffeine versus water conditions (Figure 5I). Strikingly, these genes were strongly related to metabolic processes (Figure 5J), suggesting that caffeine plays a role in re-setting histone acetylation profiles of metabolic genes in bulk tissue (i.e., presumably in non-neurons), so that they became highly inducible under learning conditions (when metabolic support is most required). We further confirmed these findings by integrating these RNA-Seq data with the H3K27ac CUT&Tag-Seq data sets using all cells (Supplemental Figure 7, C–E).

## Discussion

Caffeine is the most widely consumed psychoactive drug in the world. However, there is a striking mismatch between the epidemiological evidence associating the regular intake of caffeine with benefits for chronic brain disorders and the molecular clarification of the effect of caffeine on brain function. In fact, the majority of molecular and neurophysiological studies have explored the effects of acute rather than repeated exposure to caffeine, which have been documented to differentially affect brain function (38–40). Herein, using a combination of non-hypothesis-driven omics approaches, we show that, in the bulk tissue analysis, chronic caffeine treatment reduced metabolic processes related to lipids, mitochondria, and translation in the mouse hippocampus, some of which were identified at the different molecular levels analyzed, i.e. epigenome, transcriptome, proteome, and metabolome. In sharp contrast to what was observed in bulk tissue, we found that caffeine induced a neuron-autonomous epigenomic response related to synaptic plasticity activation. These data were corroborated by the fact that caffeine treatment induced an increase in glutamatergic synapse proteins in the hippocampus and ultimately enhanced transcriptomic regulations in response to learning. Overall, our data prompt consideration of the concept that in the brain, regular caffeine intake promotes more efficient encoding of experience-related events. By coordinating epigenomic changes in neuronal and non-neurons, regular caffeine intake promotes fine-tuning of metabolism in resting conditions, likely improving neuronal activity in response to learning.

A major finding of this study is that 2-week exposure to caffeine induced a pronounced decrease in histone acetylation (H3K27ac and H3K9/K14ac) in genes associated with several metabolic processes in the dorsal hippocampus in basal/resting conditions. H3K27ac-depleted genes had decreased *z* scores in the caffeine-treated group, additionally suggesting a mild effect on gene transcription. These data were supported by a metabolomic study indicating a global decrease in metabolites and lipids in the same experimental conditions, as well as by a proteomic analysis suggesting a decrease in energy metabolism with the reduction in levels of several proteins involved in mitochondrial activity (e.g., NDUFA3 and MPC1). These chronic changes were to some extent related to acute caffeine treatment, as a few genes were similarly affected following 24-hour and 2-week caffeine treatment, in line with Yu et al. (41), but the changes were mainly associated with long-term exposure to caffeine, as found for, e.g., the *Cyp51* gene, encoding a protein involved in cholesterol and lipid metabolism. In accordance with these findings, we found that 14 of 49 downregulated hippocampal proteins remained altered 2 weeks after caffeine withdrawal, indicating that the effects of chronic caffeine exposure persisted, as previously suggested (42). Among these, we identified ACSL4 and GNA14, which are involved in the cellular synthesis of fatty acids/lipids; and IGF2 receptor and ITPR3, involved in insulin-dependent regulation. Importantly, these data are in line with and bring molecular support to recent functional magnetic resonance imaging data showing that habitual coffee drinkers exhibit decreased brain functional connectivity at rest (43). As bulk hippocampal tissue was investigated, the cell types underlying such a metabolic decrease are yet to be identified. Independent IPA analysis of our 2 sets of epigenomic data (ChIP-Seq on bulk hippocampal tissue and CUT&Tag-Seq on dissociated hippocampal cells [“all cells”]) particularly indicated 3 common upstream regulators: TCF7L2, MKNK1, and NFASC. In the mouse brain, these genes are predominantly expressed by non-neuronal cells: TCF7L2 is preferentially expressed by newly formed oligodendrocytes and astrocytes; NFASC in newly formed oligodendrocytes; while MKNK1 is enriched in microglia (see https://www.brainrnaseq.org/). IPA analysis of “all cells” CUT&Tag-Seq data further highlighted the involvement of GLI1 and SOX2, which are both particularly enriched in astrocytes. These observations strongly support that the basal/resting signatures elicited by chronic caffeine intake may rely on non-neuronal, likely glial, responses.

We showed that, concomitant with the deacetylation process observed in the bulk hippocampus, chronic caffeine induced a neuron-autonomous epigenomic response using both active (H3K27ac) and repressive (H3K27me3) marks: acetylation of H3K27 was enriched while its trimethylation was depleted at genes related to membrane potential, potassium ion regulation, and learning and memory processes. This suggests that the overall chronic caffeine effect positively regulated neuronal activity and synaptic transmission. Proteomic studies supported this argument, as a series of proteins identified as upregulated were related to the glutamatergic synapse. It is interesting to note that 73 of 130 upregulated proteins — some of them related to the synapse — remained elevated even after a 2-week caffeine withdrawal, revealing a long-lasting effect of chronic caffeine intake on neurons. Integration of epigenomic and proteomic data particularly pointed toward CRTC1, which is known to act as a coincidence sensor of calcium and cAMP signals in neurons, triggering a transcriptional response involved in late-phase LTP maintenance at hippocampal synapses (44). We further observed that chronic caffeine intake affected the learning/training-induced transcriptome by significantly enhancing the number of differentially regulated genes. Integration of these data with the epigenomic data led to identification of a group of 121 genes related to metabolic processes that, along with being overactivated in caffeine-treated mice in learning conditions, were deacetylated, with decreased overall expression, in resting conditions (*z* score). This suggests that the resting-state effect of caffeine in non-neuronal/glial cells might be a prerequisite for the robust activation of metabolic pathways, which then improves the quality and precision of learning-associated processes, in line with its cognition-enhancing function.

Thus, a major overall conclusion of the present study is that regular caffeine intake can exert a long-term effect on neuronal activity/plasticity in the adult brain through concerted actions on the epigenome, transcriptome, proteome, and metabolome, ultimately fine-tuning metabolism-related processes and simultaneously priming activity-dependent regulations for a more efficient response to stimulation. In other words, in non-neurons, caffeine decreased the “-omic” activities under basal conditions and improved the signal-to-noise ratio during information encoding in brain circuits, thus bolstering the salience of information in brain circuits. Remarkably, the dual and opposing effects of caffeine under resting conditions and upon brain activation are in line with human brain imaging studies: under basal conditions, caffeine increases brain entropy (45) and decreases functional connectivity (46), whereas it increases blood oxygen level–dependent (BOLD) activation in the frontopolar and cingulate cortex in a verbal working memory task (47), reflecting increased processing potential. Additionally, neurophysiological studies on the putative targets of caffeine — adenosine receptors (ARs) — are in line with this dual role of caffeine, as shown by the contrasting effects of A_2A_R enhancing glutamate release and A_1_R mediating inhibition of basal synaptic transmission (48), which is also controlled by A_2A_R (49). Our data also show that the amplitude of the transcriptomic effects of caffeine was far greater when neuronal networks were activated during the learning process, as noted by others when studying the effect of caffeine on gene expression in the basal ganglia (50). This might particularly relate to a “priming” of neuronal activity, which would favor the rise of activity-dependent responses, as has been suggested for the mechanism of action of histone deacytelase inhibitors (51). How caffeine coordinates these epigenomic responses in the different cell types is an interesting question that we are currently pursuing.

Finally, the present study highlights the molecular effect of caffeine in the homeostatic brain, a subject that warrants further investigation, particularly regarding the differential mechanisms operating at the cellular level that modulate resting and active physiological brain activity. Additionally, our data could have far-reaching implications for the study of synaptic dysregulation, such as Alzheimer’s disease: while caffeine exhibits normalizing properties in models of such disorders (52–54), the cell-specific molecular mechanisms remain to be uncovered. On the opposite end of the allostatic brain spectrum (55), caffeine has been suggested to affect synaptic fate in brain development (56, 57), but the involvement of neuronal versus non-neuronal mechanisms remains ill defined. It is therefore particularly relevant and important to address, on a larger scale, the integrated actions of caffeine in neuronal versus non-neurons in the immature, homeostatic, and aging brain.

## Methods

### Animals.

Male C57BL6/J mice (Charles River Laboratories) were housed in a pathogen-free facility (University of Lille). Mice were housed 5–6 per cage (GM500, Tecniplast) and maintained under conditions controlled for temperature (22°C) and light (12-hour light/12-hour dark cycle), with ad libitum access to food and water.

### Caffeine treatment.

Two 3-month-old mice were randomly assigned to the 2 following experimental groups: water (control) and caffeine. Caffeine solutions were protected from light in dark bottles and changed weekly. Treatment started at 8–9 weeks of age and lasted for 2 weeks. The chronic caffeine treatment in mice was set in order to mimic the usual dose range of caffeine consumption in humans. The selected caffeine dose of 0.3 g/L p.o., administered through drinking water, has been previously shown to provide a significant benefit in mouse models of neurodegenerative disease (53, 54, 58). Regarding the comparison of caffeine exposure for 2 weeks versus 24 hours versus caffeine removal, we proceeded with additional experimental groups as follows: (i) 6 animals were given water while 6 were treated with caffeine for 2 weeks and returned to water for 2 additional weeks (caffeine withdrawal group); (ii) at the same time that the latter group of animals resumed water exposure, 6 additional animals were treated with caffeine for 2 weeks (chronic caffeine); (iii) a last group of 6 animals was given water and only treated with caffeine for 24 hours before sacrifice. All animals were then sacrificed on the same day the dorsal hippocampus was sampled and stored as indicated below and used for proteomics and qPCR analysis.

### Quantitative determination of caffeine and metabolites in brain samples.

Brain tissues from the water and caffeine groups were used to assess concentrations of caffeine and its metabolites (paraxanthine, theobromine, and theophylline). Samples were weighed, and 1 mL of 1% formic acid (FA) solution was added to each sample. To determine the recovery rate, control samples were spiked with a mixture of caffeine, paraxanthine, theobromine, and theophylline (10 μM each). The tissues were lysed using 7 mm stainless steel beads and Tissue Lyser LT (QIAGEN) for 8 minutes at 50 strokes/min, then treated with an ultrasonic bath for 5 minutes and subsequently centrifuged for 15 minutes at 23,000*g* and 4°C. The supernatants were transferred to Amicon Ultra 2-mL 3K Centrifugal Filter units (Merck). The remaining pellets were subjected to the same protocol of tissue disruption and centrifugation using 1 mL of acidified water (1% FA). Amicon filters containing the combined supernatants from the 2-fold extraction process were centrifuged for 140 minutes at 7500*g* and 23°C. Filtrates were used for liquid chromatography–MS (LC-MS) analysis. Samples were separated by use of a Dionex UltiMate 3000 HPLC system with an integrated variable wavelength detector, set at 280 nm, and equipped with a C18 column (EC Nucleodur C18 Gravity column, 2 mm ID × 50 mm, 3 μm, Macherey-Nagel). Samples (5 μL) were injected at flow rate of 300 μL/min. A solvent gradient was run from 90% solution A (water containing 0.2% FA and 2 mM ammonium acetate) and 10% solution B (methanol containing 2 mM ammonium acetate) to 50% A and 50% B over 10 minutes.

The eluate was analyzed with a coupled mass spectrometer (ESI-micrOTOF-Q, Bruker Daltonics). Data were acquired in positive full scan MS mode with a scan range *m/z* of 50–1000. Identification and quantification of the xanthine derivatives were performed using Bruker Daltonics data analysis software. The limit of detection was 5 nM for caffeine and 10 nM for its metabolites (paraxanthine, theobromine, and theophylline).

### Learning activation in the MWM.

An Atlantis MWM tank was placed in a room with several visual extra-maze cues. Water opacified with powdered chalk (Blanc de meudon) was maintained at a temperature of 21°C. Mice from the water (control) and caffeine groups were habituated to the setup for 2 consecutive days (habituations 1 and 2). During habituation 1, mice were allowed to discover the pool filled with water with a depth of 5 cm and a visible platform for 60 seconds. During habituation 2, mice were allowed to swim in the pool filled with water in the absence of the platform for 60 seconds. The following 3 days (acquisition days 1–3), mice were trained to locate the platform beneath the opacified water using the spatial cues present in the room. On each acquisition day, mice performed 4 trials each with a maximum duration of 60 seconds. Each trial was terminated when the mouse reached the platform or after the 60 seconds. Mice failing to find the platform were gently guided to the platform and allowed to stay for 8 –10 seconds. During the training days, mice were subjected to the MWM test in random order, so that they were tested at different times of the day. All MWM evaluations of caffeine- or water-treated mice were performed by an experimenter blinded to the mouse treatments.

### Sacrifice and brain tissue preparation.

For transcriptomic analysis, all mice were killed by cervical dislocation within a 2 hour period of time — taking into account a 1 hour delay after the last training session for the learning group. Freshly dissected tissues were immediately frozen in liquid nitrogen and kept at –80°C until RNA extraction. Similar sacrifice procedures were used for animals used for proteomic and qPCR analyses. For molecular MALDI imaging experiments, mice were deeply anesthetized with sodium pentobarbital (50 mg/kg, i.p.), then transcardially perfused with cold NaCl (0.9%). Brains were collected, frozen on dry ice, and stored at –80°C until use.

### RNA-Seq analysis.

Total RNA was extracted from dorsal hippocampal tissues using TRIzol reagent (Invitrogen; *n* = 4/group). Freshly dissected tissue was chopped, homogenized in 300 μL TRIzol reagent, and frozen (20 minutes at –80°C), followed by 3 minutes of centrifugation at 14,000*g* before chloroform/isoamyl extraction. The supernatant was used to precipitate RNA with isopropanol and RNase-free glycogen (30 minutes at 4°C). The pellet was washed once with 70% ethanol and resuspended in Milli-Q water. A new RNA precipitation was performed with 100% ethanol and 3 M sodium acetate (overnight at –20°C). After 2 further 70% ethanol washes, the pellet was air dried and resuspended in 30 μL nuclease-free Milli-Q water and heated for 6 minutes at 50°C, and RNA quantification was performed. RNA-Seq libraries (*n* = 4/group) were generated from 500 ng total RNA using Illumina TruSeq Stranded mRNA Library Prep Kit v2. Briefly, following purification with poly-T oligo–attached magnetic beads, the mRNA was fragmented using divalent cations at 94°C for 2 minutes. The cleaved RNA fragments were copied into first-strand cDNA using reverse transcriptase and random primers. Strand specificity was achieved by replacing dTTP with dUTP during the second-strand cDNA synthesis by DNA polymerase I and RNase H. Following addition of a single “A” base and subsequent ligation of the adapter on double-stranded cDNA fragments, the products were purified and enriched with PCR (30 seconds at 98°C [10 seconds at 98°C, 30 seconds at 60°C, 30 seconds at 72°C] × 12 cycles; 5 minutes at 72°C) to create the cDNA library. Surplus PCR primers were further removed by purification using AMPure XP beads (Beckman Coulter), and the final cDNA libraries were checked for quality and quantified using capillary electrophoresis. Next generation sequencing (NGS) was performed on the Illumina Genome HiSeq 4000 as single-end 50 base reads following Illumina’s instructions. Reads were mapped onto the mm10 assembly of the *Mus musculus* genome using STAR v2.5.3a (59) and Bowtie 2 aligner v2.2.8 (60). Only uniquely aligned reads were kept for further analyses. Quantification of gene expression was performed using HTSeq-count v0.6.1p1 (61) and gene annotations from Ensembl release 90 and “union” mode. Read counts were normalized across libraries with the method proposed by Anders et al. (62). Comparisons of interest were performed using the test for differential expression proposed by Love (63) and implemented in the DESeq2 Bioconductor library (v1.16.1). Resulting *P* values were adjusted for multiple testing using the Benjamini-Hochberg method (64).

### ChIP.

Freshly dissected tissue was chopped with a razor blade and rapidly incubated in 1.5 mL PBS containing 1% formaldehyde for 10 minutes at room temperature. To stop fixation, glycine was added (0.125 M final concentration). Dorsal hippocampi from 4 mice were pooled per sample, and 2 biological replicates per condition were used for the ChIP-Seq. Tissue samples were then processed as described in Chatterjee et al. (34) and sonicated using the Diagenode Bioruptor (30 seconds ON — 30 seconds OFF at high power × 35 cycles). Sonicated chromatin was centrifuged 10 minutes at 14,000*g*, and the supernatant was collected and diluted 1:10 in ChIP dilution buffer (0.01% SDS, 1.1% Triton X-100, 1.2 mM EDTA, 16.7 mM Tris-Cl, pH 8.1, 167 mM NaCl). A fraction of the supernatant (50 μL, 10%) from each sample was saved before immune precipitation for “total input chromatin.” Supernatants were incubated overnight (4°C) with 1/1000 primary antibodies against H3K9/14ac (Diagenode, C15410200) and H3K27ac (Abcam, ab4729), followed by protein A Dynabeads (Invitrogen) for 2 hours at room temperature. After several washes (low-salt, high-salt, LiCl, and TE buffers), the resulting DNA-protein complexes were eluted in 300 μL elution buffer (1% SDS, 0.1 M NaHCO_3_). The crosslinking was reversed (overnight at 65°C), and the DNA was subsequently purified with RNase (30 minutes at 37°C) and proteinase K (2 hours at 45°C). DNA from the immunoprecipitated and input samples was isolated using Diagenode MicroChIP DiaPure columns with 20 μL nuclease-free milliQ water in low-binding tubes. ChIP samples were further purified by the Genomeast Platform using Agencourt AMPure XP beads (Beckman Coulter) and quantified using Qubit (Invitrogen).

### ChIP-Seq libraries and sequencing.

ChIP-Seq libraries and NGS were prepared from 2–10 ng double-stranded, purified DNA using the MicroPlex Library Preparation kit v2 (C05010014, Diagenode), according to the manufacturer’s instructions. DNA was first repaired and yielded molecules with blunt ends. Next, stem-loop adapters with blocked 5′ ends were ligated to the 5′ end of the genomic DNA (gDNA), leaving a nick at the 3′ end. The adapters cannot ligate to each other and do not have single-strand tails; thus, nonspecific background is avoided. In the final step, the 3′ ends of the gDNA were extended to complete library synthesis, and Illumina-compatible indexes were added through PCR amplification (4+7 cycles). Amplified libraries were purified and size selected using Agencourt AMPure XP beads to remove unincorporated primers and other reagents. Prior to analyses, DNA libraries were checked for quality and quantified using a 2100 Bioanalyzer (Agilent). The libraries were loaded in the flow cell at a concentration of 8 pM, and clusters were generated using cBot and sequenced using Illumina HiSeq 4000 technology as single-end 50 base reads following Illumina’s instructions. Basecalls were performed using HiSeq Control Softwares Real Time Analysis (RTA) and CASAVA.

### ChIP-Seq analyses.

Sequenced reads were mapped to the mm10 *Mus musculus* genome assembly using Bowtie v1.0.0 with the following parameters “-m1-strata-best-y-l40.” SAMtools merge v1.3.1 (65) was used to combine biological replicates by condition. Then, BEDTools intersect v2.26.0 (66) was used to remove reads located within ENCODE-blacklisted regions (see below). SICER (SICER-df.sh) v1.1 (67) was used to detect differentially bound regions on the pools of biological replicates using the following parameters: Species: mm10, Effective genome size as a fraction of reference genome: 0.74, Threshold for redundancy allowed for treated reads: 1, Threshold for redundancy allowed for WT reads: 1, Window size: 200 bps, Fragment size: 200 bps, Gap size: 600 bps, FDR for identification of enriched islands: 1 × 10^–2^, FDR for identification of significant changes: 1 × 10^–2^. Finally, differentially bound regions were annotated with respect to the closest gene using Homer annotatePeaks.pl v4.11.1 (68). An FDR of 1 × 10^–5^ was used in differential analyses (caffeine vs. control).

### Neuron and “all cell” isolation.

Neuron and “all cell” suspensions were obtained from mouse hippocampus chronically treated with caffeine or water (control). For this, we used a Neural Tissue Dissociation (Miltenyi Biotec, 130-092-628) and Neuron Isolation Kits (Miltenyi Biotec, 130-115-389), following the manufacturer’s instructions, with some adaptations. Briefly, 2 mouse hippocampi were pooled per sample and harvested in a preheated buffer solution containing papain. This was followed by series of manual mechanical dissociations, using scissors and fire-polished Pasteur pipettes of descending diameter, and incubations at 37°C under slow rotation. The solution was then filtered (50 μm) and centrifuged (10 minutes, 300*g*, at room temperature) and myelin was removed using a Myelin Removal Beads II kit (Miltenyi Biotec, 130-096-733), incubating for 15 minutes at 4°C, centrifuging (10 minutes, 300*g*, at 4°C), and filtering the sample through MS columns (Miltenyi Biotec, 130-042-201) placed in a MiniMACS Separator (Miltenyi Biotec, 30-042-102) to collect the myelin-depleted flow-through, free of cell debris. The “all cells” suspension was collected at this point and quantified using the TC20 Automated Cell Counter (Bio-Rad, 1450102) to obtain a total of 70,000 cells per sample. With the remainder of the samples, we proceeded with neuronal isolation according to manufacturer’s instructions, finally depleting the samples through MS columns to collect the flow-through enriched in neurons. The samples were quantified, and 70,000 cells per sample were taken for CUT&Tag experiments.

### CUT&Tag.

Having isolated all cells and neuronal populations, we proceeded with the CUT&Tag method to assess their genome-wide H3K27ac and H3K27me3 chromatin state. The protocol was adapted from that described by Kaya-Okur et al. (31). The method is based on digitonin-induced cell permeabilization (MilliporeSigma, 300410-250MG) and concanavalin A–coated magnetic beads (Cell Signaling Technology, 93569S) immobilization. This was followed by overnight incubation at 4°C with primary antibodies against H3K27ac (Abcam, ab4729) and H3K27me3 (Diagenode, C15410195), and 1-hour incubation with the secondary antibody (Antibodies-online, ABIN101961). Loaded Tn5 was then added (Diagenode, C01070001), and cleaved DNA was extracted using a MinElute PCR Purification Kit (QIAGEN, 28004). Library preparation was conducted using Nextera primers (Illumina, FC-131-2001) and post-PCR cleanup using SPRI bead slurry (Beckman Coulter, B23317). Concentration of the collected DNA was achieved by Qubit (Invitrogen, Q32851). Two biological replicates were used per group, and rabbit IgG (Diagenode, C15410206) was used as control.

### CUT&Tag analyses.

Reads (paired-end) were mapped to the *Mus musculus* genome (assembly mm10) using Bowtie2 (60) v2.2.8 with default parameters except for “–end-to-end-very-sensitive-no-mixed –no-discordant-I10-X700.” Prior to peak calling, reads with mapping quality below 30 were removed using SAMtools v1.13 (65) with the command line “samtools view-b-q30.” Then, reads falling into ENCODE-blacklisted regions v2 (69) were removed using BEDTools intersect v2.30.0 (66). Biological replicates were pooled (*n* = 2) using SAMtools merge v1.13 (65). Then, peak calling was done with SICER v1.1 (68) with the following parameters: Window size: 200 bps; Gap size: 800 (H3K27ac) and 1200 (H3K27me3). Detected peaks were combined to get the union of all peaks using the tool BedTools merge v2.30.0 (66). Differentially bound regions were detected used SICER v1.1 and annotated relative to genomic features using Homer v4.11.1 (70). An FDR < 1 × 10^–5^ was used for further analyses (caffeine vs. control) in all cells and neuron-enriched population.

### MS proteomic analysis.

Proteins (100 μg) from dorsal hippocampus (*n* = 3/group) were digested with trypsin by the filter-aided sample preparation (FASP) method. Peptides were fractioned with increasing concentrations (7.5%, 12.5%, 17.5%, and 50%) of acetonitrile on a High pH Reversed-Phase Peptide Fractionation Kit (Thermo Fisher Scientific). Eluents were dried by vacuum centrifugation and resolved in 0.1% FA. An UltiMate 3000 RSLCnano System (Thermo Fisher Scientific) was used for separation of the eluents. Peptides were automatically fractionated onto a commercial C18 reversed-phase column (75 μm × 500 mm, 2 μm particle, PepMap100 RSLC column, Thermo Fisher Scientific, temperature 55°C). Trapping was performed during 4 minutes at 5 μL/min, with solvent A (98% H_2_O, 2% acetonitrile, and 0.1% FA). The peptides were eluted using 2 solvents: A (0.1% FA in water) and B (0.1% FA in acetonitrile) at a flow rate of 300 nL/min. Gradient separation was 3 minutes at 3% B; 170 minutes from 3% to 20% B; 20 minutes from 20% B to 80% B; and maintained for 15 minutes at 80% B. The column was equilibrated for 17 minutes with 3% B prior to the next sample analysis. The eluted peptides from the C18 column were analyzed by a Q Exactive instrument (Thermo Fisher Scientific). The electrospray voltage was 1.9 kV, and the capillary temperature was 275°C. Full MS scans were acquired in an Orbitrap mass analyzer over a *m/z* range of 400–1200 with a 70,000 (*m/z* 200) resolution. The target value was 3.00 × 10^6^. The 15 most intense peaks with charge state between 2 and 5 were fragmented in the higher-energy collision-activated dissociation cell with normalized collision energy of 27%, and tandem mass spectrum was acquired in the Orbitrap mass analyzer with a 17,500 (*m/z* 200) resolution. The target value was 1.00 × 10^5^. The ion selection threshold was 5.0 × 10^4^ counts, and the maximum allowed ion accumulation times were 250 ms for full MS scans and 100 ms for tandem mass spectrum. Dynamic exclusion was set to 30 seconds.

### Proteomic data analysis.

Raw data collected during nanoLC–MS/MS analyses were processed and converted into a *.mgf peak list format with Proteome Discoverer 1.4 (Thermo Fisher Scientific). MS/MS data were analyzed using search engine Mascot (version 2.4.0, Matrix Science) installed on a local server. Searches were performed with a tolerance on mass measurement of 10 ppm for precursor and 0.02 Da for fragment ions, against a composite target-decoy database (17125*2 total entries) built with a *Mus musculus* SwissProt database (taxonomy 10090, November 2019, 17,007 entries) fused with the sequences of recombinant trypsin and a list of classical contaminants (118 entries). Cysteine carbamidomethylation, methionine oxidation, protein N-terminal acetylation, and cysteine propionamidation were searched as variable modifications. Up to one missed trypsin cleavage was allowed. For each sample, peptides were filtered out according to the cutoff set for protein hits with 1 or more peptides longer than 9 residues and a 1% false positive rate.

### MALDI MS imaging of lipids and metabolites.

Hippocampal sections (10 μm) were collected at bregma –1.7 mm using a CM3050 Cryostat (Leica Microsystems; *n* = 6 per experimental group) and then mounted on indium tin oxide–coated slides for MALDI-MSI and on SuperFrost slides (Thermo Fisher Scientific) for histological analysis. In order to monitor analytical reproducibility, biological replicates were used for each group. For MALDI-MSI, 1,5-diaminonaphthalene (1,5-DAN) matrix at 10 mg/mL in acetonitrile:H_2_O (1:1, v/v) was used in negative ion mode, whereas 2,5-dihydroxybenzoic acid (2,5-DHB) matrix at 40 mg/mL in methanol:H_2_O with 0.1% trifluoroacetate (1:1, v/v) was used in positive ion mode. A uniform layer of matrix was deposited on brain tissue sections using an HTX TM-Sprayer device (HTX Technologies). Lipid and metabolite imaging was performed on a solariX 7T MALDI-FTICR instrument (Bruker Daltonics) equipped with a SmartBeam-II laser and controlled using FtmsFlexControl 2.1.0 software (Bruker Daltonics). Data sets were recorded in full-scan negative- or positive-ion mode using an online calibration from *m/z* of 100 to 1000, at a spatial resolution of 35 μm for the hippocampus. MSI data were acquired from each tissue section as well as matrix-adjacent control areas in order to check for analyte delocalization eventually occurring during sample preparation. All data processing, visualization, and quantification were performed using Multimaging 1.1.9 software (ImaBiotech). For statistical analysis, SCiLS Lab 2015 software was used to perform PCA, with a Student’s *t* test assumed significance value of *P* < 0.05. These analyses were done for both positive- and negative-ion mode, and the significant results were grouped together. Annotation of the discriminant *m/z* was done based on experimental accurate (*m/z*) mass and by using the METLIN library (https://metlin.scripps.edu/landing_page.php?pgcontent=mainPage) and Human Metabolome Database (HMDB; http://www.hmdb.ca/) with 10 ppm delta error. These online databases are linked to KEGG (http://www.genome.jp/kegg/), PubChem (https://pubchem.ncbi.nlm.nih.gov/) and LIPID MAPS (http://www.lipidmaps.org/), which were used for further investigations. After MSI data acquisition, any residual matrix on the tissue sections was removed with a 100% methanol washing. Tissue sections were then stained with Nissl dye, and high-definition histological images were acquired using a Panoramic digital slide scanner (3DHISTECH) and then loaded in Multimaging software to perform the high-definition overlays with convoluted molecular images, improving molecular image resolution.

### RNA extraction and qPCR.

Total RNA was extracted from dorsal hippocampi and purified using an RNeasy Lipid Tissue Mini Kit (QIAGEN). Total RNA (500 ng) was reverse transcribed using an Applied Biosystems High-Capacity cDNA Reverse Transcription Kit. qPCR was performed on a StepOne device using Taqman Gene Expression Master Mix (Thermo Fisher Scientific), following the manufacturer’s recommendations. Expression levels of the following genes were evaluated by the comparative CT method (2–ΔCT) using the following Taqman probes: *Cyp51* ID: Mm00490968_m1, *Spice1* ID: Mm00519954_m1, *Nadk2* ID: Mm01297768_m1, *Pbx1* ID: Mm04207617_m1, *Ppia* ID: Mm02342430.

### Pathway analysis of epigenomic data.

ChIP-Seq and CUT&Tag data from hippocampus of water- and caffeine-treated mice were uploaded to IPA software (QIAGEN). A *P* value less than 0.05 with Student’s *t* test was set as threshold, and an IPA of ChIP-Seq, proteomic, and metabolomic data was performed using the core analysis function, including canonical pathways, upstream regulators, diseases, biological functions, and molecular networks filtered by terms associated with “Central Nervous System.”

### Statistics.

This omics study included various statistical approaches detailed in the appropriate subsections in Method. All data needed to evaluate the conclusions are provided herein or in the supplemental material. Sequencing data that support the findings of this study have been deposited in the NCBI’s Gene Expression Omnibus (GEO) database (GSE167123). The number of biologically independent experiments, sample size, *P* values, and statistical tests are indicated in the main text and/or figure legends. The significance level was set at *P* < 0.05, unless otherwise stated in the figure legend.

### Study approval.

All experimental protocols were approved by the local Animal Ethical Committee (agreement 12787-2015101320441671v9 from CEEA75, Lille). All procedures complied with European standards for the care and use of laboratory animals.

## Author contributions

IP, LC, CM, LP, LB, VBS, RAB, NS, JSA, ALB, and DB conceptualized the study. IP, LC, CM, LP, AP, HD, SLG, CEM, EF, KC, SE, J Seguin, J Stauber, ON, JMS, and ASL designed the methodology. IP, LC, CM, LP, AP, HD, SLG, MS, EMM, EF, KC, VGM, BT, SE, ES, J Stauber, RAB, NS, LT, and IG performed the investigations. IP, LC, CM, LP, HD, SLG, CEM, DV, J Seguin, RAB, NS, JSA, ALB, DB, ON, and JMS performed data analysis. RAB, NS, JSA, ALB, and DB supervised the study. LC, IP, CM, LP, DV, LVL, RAC, RAB, NS, JSA, ALB, and DB wrote the original draft of the manuscript. LC, IP, CM, CEM, TL, LVL, LB, VBS, RAC, NS, JSA, ALB, and DB wrote, reviewed, and edited the manuscript.

## Supplementary Material

Supplemental data

Supplemental table 1

Supplemental table 2

Supplemental table 3

Supplemental table 4

Supplemental table 5

Supplemental table 6

Supplemental table 7

Supplemental table 8

Supplemental table 9

## Version 1 05/10/2022

In-Press Preview

## Version 2 06/15/2022

Electronic publication

## References

[1] Smith A (2002). Effects of caffeine on human behavior. Food Chem Toxicol.

[2] Kim Y (2019). Coffee consumption and all-cause and cause-specific mortality: a meta-analysis by potential modifiers. Eur J Epidemiol.

[3] Loftfield E (2018). Association of coffee drinking with mortality by genetic variation in caffeine metabolism: findings from the UK Biobank. JAMA Intern Med.

[4] Freedman ND (2012). Association of coffee drinking with total and cause-specific mortality. N Engl J Med.

[5] Flaten V (2014). From epidemiology to pathophysiology: what about caffeine in Alzheimer’s disease?. Biochem Soc Trans.

[6] Cunha RA (2016). How does adenosine control neuronal dysfunction and neurodegeneration?. J Neurochem.

[7] Cellai L (2018). The adenosinergic signaling: a complex but promising therapeutic target for Alzheimer’s disease. Front Neurosci.

[8] Wright GA (2013). Caffeine in floral nectar enhances a pollinator’s memory of reward. Science.

[9] Marques S (2011). Modulating Alzheimer’s disease through caffeine: a putative link to epigenetics. J Alzheimers Dis.

[10] Angelucci MEM (2002). Effects of caffeine on learning and memory in rats tested in the Morris water maze. Braz J Med Biol Res.

[11] Borota D (2014). Post-study caffeine administration enhances memory consolidation in humans. Nat Neurosci.

[12] Lopes JP (2019). The physiological effects of caffeine on synaptic transmission and plasticity in the mouse hippocampus selectively depend on adenosine A(1) and A(2A) receptors. Biochem Pharmacol.

[13] Costenla AR (2010). Caffeine, adenosine receptors, and synaptic plasticity. J Alzheimers Dis.

[14] Simons SB (2011). Caffeine-induced synaptic potentiation in hippocampal CA2 neurons. Nat Neurosci.

[15] Lao-Peregrín C (2017). Caffeine-mediated BDNF release regulates long-term synaptic plasticity through activation of IRS2 signaling. Addict Biol.

[16] Blaise JH (2018). Caffeine consumption disrupts hippocampal long-term potentiation in freely behaving rats. Physiol Rep.

[17] Watanabe Y, Ikegaya Y (2017). Caffeine increases hippocampal sharp waves in vitro. Biol Pharm Bull.

[18] Hanajima R (2019). Effect of caffeine on long-term potentiation-like effects induced by quadripulse transcranial magnetic stimulation. Exp Brain Res.

[19] Kerkhofs A (2017). Caffeine controls glutamatergic synaptic transmission and pyramidal neuron excitability in human neocortex. Front Pharmacol.

[20] Fredholm BB (1999). Actions of caffeine in the brain with special reference to factors that contribute to its widespread use. Pharmacol Rev.

[21] Heintzman ND (2007). Distinct and predictive chromatin signatures of transcriptional promoters and enhancers in the human genome. Nat Genet.

[22] Hnisz D (2013). Super-enhancers in the control of cell identity and disease. Cell.

[23] Parker SCJ (2013). Chromatin stretch enhancer states drive cell-specific gene regulation and harbor human disease risk variants. Proc Natl Acad Sci U S A.

[24] Karmodiya K (2012). H3K9 and H3K14 acetylation co-occur at many gene regulatory elements, while H3K14ac marks a subset of inactive inducible promoters in mouse embryonic stem cells. BMC Genomics.

[25] Sánchez-Sarasúa S (2022). IRS1 expression in hippocampus is age-dependent and is required for mature spine maintenance and neuritogenesis. Mol Cell Neurosci.

[26] Koopmans F (2019). SynGO: an evidence-based, expert-curated knowledge base for the synapse. Neuron.

[27] Sheng M, Kim E (2000). The Shank family of scaffold proteins. J Cell Sci.

[28] Mundel P (1997). Synaptopodin: an actin-associated protein in telencephalic dendrites and renal podocytes. J Cell Biol.

[29] Parra-Damas A (2017). CRTC1 function during memory encoding is disrupted in neurodegeneration. Biol Psychiatry.

[30] Wang L, Walter P (2020). Msp1/ATAD1 in protein quality control and regulation of synaptic activities. Annu Rev Cell Dev Biol.

[31] Kaya-Okur HS (2019). CUT&Tag for efficient epigenomic profiling of small samples and single cells. Nat Commun.

[32] Han S (2010). Regulation of dendritic spines, spatial memory, and embryonic development by the TANC family of PSD-95-interacting proteins. J Neurosci.

[33] Parra-Damas A (2017). CRTC1 mediates preferential transcription at neuronal activity-regulated CRE/TATA promoters. Sci Rep.

[34] Chatterjee S (2018). Reinstating plasticity and memory in a tauopathy mouse model with an acetyltransferase activator. EMBO Mol Med.

[35] Martínez G (2016). Regulation of memory formation by the transcription factor XBP1. Cell Rep.

[36] Cao L (2004). VEGF links hippocampal activity with neurogenesis, learning and memory. Nat Genet.

[37] Mews P (2017). Acetyl-CoA synthetase regulates histone acetylation and hippocampal memory. Nature.

[38] Jacobson KA (1996). Adenosine receptor ligands: differences with acute versus chronic treatment. Trends Pharmacol Sci.

[39] Ferré S (2008). An update on the mechanisms of the psychostimulant effects of caffeine. J Neurochem.

[40] Doepker C (2016). Caffeine: friend or foe?. Annu Rev Food Sci Technol.

[41] Yu L (2009). Uncovering multiple molecular targets for caffeine using a drug target validation strategy combining A2A receptor knockout mice with microarray profiling. Physiol Genomics.

[42] Svenningsson P (1999). The stimulatory action and the development of tolerance to caffeine is associated with alterations in gene expression in specific brain regions. J Neurosci.

[43] Magalhães R (2021). Habitual coffee drinkers display a distinct pattern of brain functional connectivity. Mol Psychiatry.

[44] Kovács KA (2007). TORC1 is a calcium- and cAMP-sensitive coincidence detector involved in hippocampal long-term synaptic plasticity. Proc Natl Acad Sci U S A.

[45] Chang D (2018). Caffeine caused a widespread increase of resting brain entropy. Sci Rep.

[46] Tal O (2013). Caffeine-induced global reductions in resting-state BOLD connectivity reflect widespread decreases in MEG connectivity. Front Hum Neurosci.

[47] Koppelstaetter F (2008). Does caffeine modulate verbal working memory processes? An fMRI study. Neuroimage.

[48] Cunha RA (2008). Different cellular sources and different roles of adenosine: A1 receptor-mediated inhibition through astrocytic-driven volume transmission and synapse-restricted A2A receptor-mediated facilitation of plasticity. Neurochem Int.

[49] Lopes LV (1999). Cross talk between A(1) and A(2A) adenosine receptors in the hippocampus and cortex of young adult and old rats. J Neurophysiol.

[50] Dassesse D (2001). Acute and chronic caffeine administration differentially alters striatal gene expression in wild-type and adenosine A(2A) receptor-deficient mice. Synapse.

[51] Burns AM, Gräff J (2021). Cognitive epigenetic priming: leveraging histone acetylation for memory amelioration. Curr Opin Neurobiol.

[52] Duarte JMN (2016). Adenosine A_1_ receptors control the metabolic recovery after hypoxia in rat hippocampal slices. J Neurochem.

[53] Laurent C (2014). Beneficial effects of caffeine in a transgenic model of Alzheimer’s disease-like tau pathology. Neurobiol Aging.

[54] Arendash GW (2006). Caffeine protects Alzheimer’s mice against cognitive impairment and reduces brain beta-amyloid production. Neuroscience.

[55] David B, Lopes LV (2021). Stabilizing synapses. Science.

[56] Silva CG (2013). Adenosine receptor antagonists including caffeine alter fetal brain development in mice. Sci Transl Med.

[57] Ferran GC (2022). Convergence of adenosine and GABA signaling for synapse stabilization during development. Science.

[58] Arendash GW (2009). Caffeine reverses cognitive impairment and decreases brain amyloid-beta levels in aged Alzheimer’s disease mice. J Alzheimers Dis.

[59] Dobin A (2013). STAR: ultrafast universal RNA-seq aligner. Bioinformatics.

[60] Langmead B, Salzberg SL (2012). Fast gapped-read alignment with Bowtie 2. Nat Methods.

[61] Anders S (2015). HTSeq — a Python framework to work with high-throughput sequencing data. Bioinformatics.

[62] Anders S, Huber W (2010). Differential expression analysis for sequence count data. Genome Biol.

[63] Love MI (2014). Moderated estimation of fold change and dispersion for RNA-seq data with DESeq2. Genome Biol.

[64] Benjamini Y, Hochberg Y (1995). Controlling the false discovery rate - a practical and powerful approach to multiple testing. J R Stat Soc Ser B-Methodological.

[65] Li H (2009). The sequence alignment/map format and SAMtools. Bioinformatics.

[66] Quinlan AR, Hall IM (2010). BEDTools: a flexible suite of utilities for comparing genomic features. Bioinformatics.

[67] Xu S (2014). Spatial clustering for identification of ChIP-enriched regions (SICER) to map regions of histone methylation patterns in embryonic stem cells. Methods Mol Biol.

[68] Zang C (2009). A clustering approach for identification of enriched domains from histone modification ChIP-Seq data. Bioinformatics.

[69] Amemiya HM (2019). The ENCODE blacklist: identification of problematic regions of the genome. Sci Rep.

[70] Heinz S (2010). Simple combinations of lineage-determining transcription factors prime cis-regulatory elements required for macrophage and B cell identities. Mol Cell.

