# Supplementary material for: Caffeine intake exerts dual genome-wide effects on hippocampal metabolism and learning-dependent transcription
Source: J Clin Invest. 2022 Jun 15;132(12):e149371. doi: 10.1172/JCI149371 (PMC9197525; doi:10.1172/JCI149371)
Supplement: Supplemental data [file jci-132-149371-s142.pdf]

Supplementary Materials for

Caffeine intake exerts dual genome-wide effects on hippocampal metabolism and learning-dependent transcription

Isabel Paiva[1†], Lucrezia Cellai[2,3†], Céline Mériaux[2,3†], Lauranne Poncelet[4†], Ouada Nebie[2,3], Jean-Michel Saliou, Anne-Sophie-Lacoste, Anthony Papegaey[2,3], Hervé Drobecq[6], Stéphanie Le Gras[7], Marion Schneider[8], Enas M. Malik[8], Christa E. Müller[8], Emilie Faivre[1,2], Kevin Carvalho[2,3], Victoria Gomez-Murcia[2,3], Didier Vieau[2,3], Bryan Thiroux[2,3], Sabiha Eddarkaoui[2,3], Thibaud Lebouvier[2,3,8], Estelle Schueller[1], Laura Tzeplaeff[1], Iris Grgurina[1], Jonathan Seguin[1], Jonathan Stauber[4], Luisa V. Lopes[9], Luc Buée[2,3], Valérie Buée-Scherrer[2,3], Rodrigo A. Cunha[10,11], Rima Ait-Belkacem[4‡], Nicolas Sergeant[2,3‡], Jean-Sébastien Annicotte[12,13‡], Anne-Laurence Boutillier[1‡*], David Blum[2,3‡*]

*Corresponding author. Email: david.blum@inserm.fr

This PDF file includes:

Figs. S1 to S6
Tables S1 to S4: only legends with excel data downloaded
Table S5
Tables S6 to S9: only legends with excel data downloaded

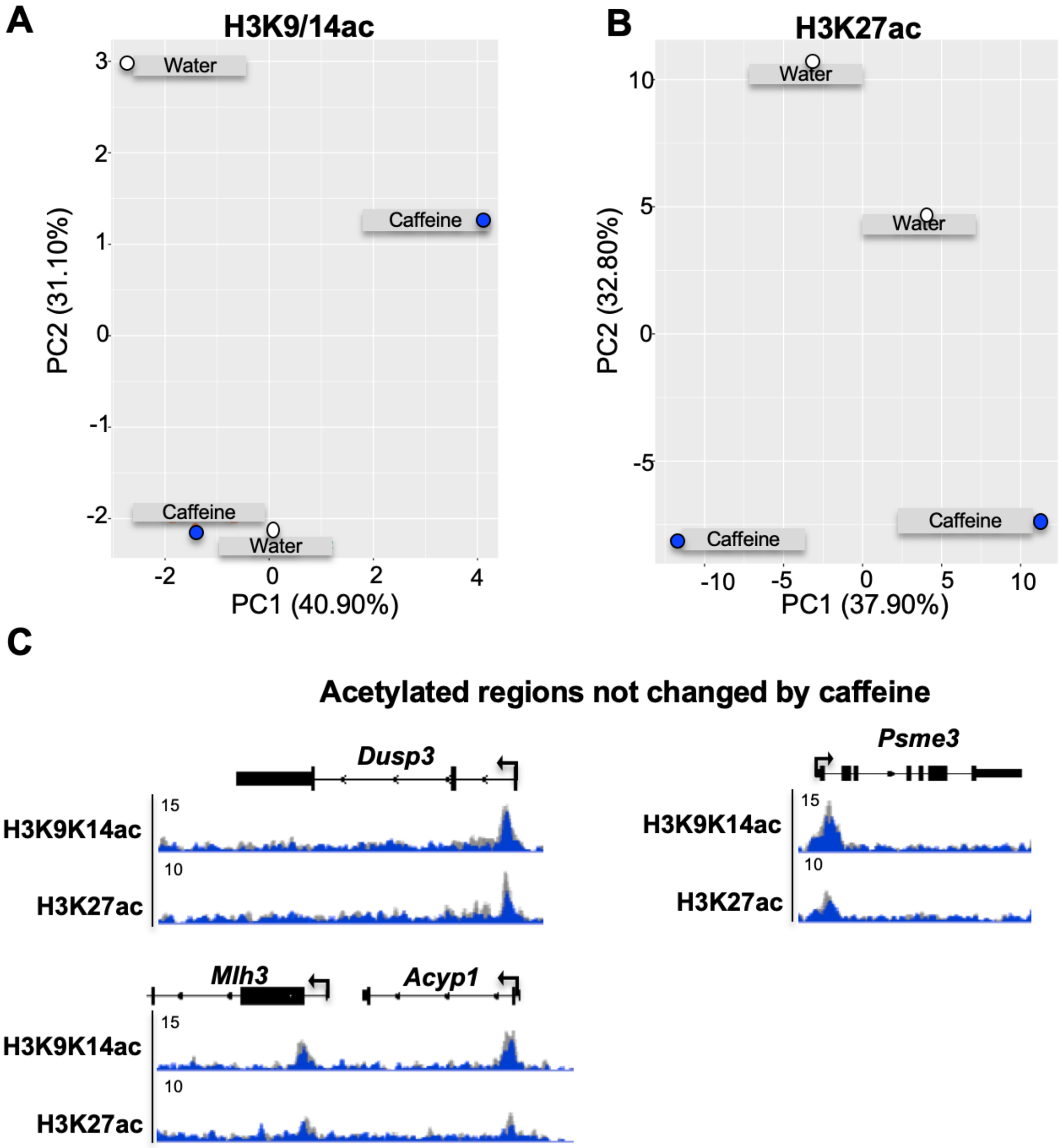

Supplemental Figure 1. ChIP-seq of bulk hippocampus of water and caffeine-treated mice. Principal Component Analysis (PCA) of control (water-treated) and caffeine-treated mice from ChIP-seq of H3K9/14ac (A) and H3K27ac (B) data (N=2). H3K27ac mark shows more robust separation between water and caffeine-treated animals. A total of 2 biological replicates were used per histone mark. (C) Genomic representation by The Integrative Genomics Viewer (IGV) of some representative H3K9/14 and H3K27 acetylated regions not altered by chronic caffeine consumption in the mouse hippocampus (*Dusp3, Psme3, Mlh3, Acyp1*).

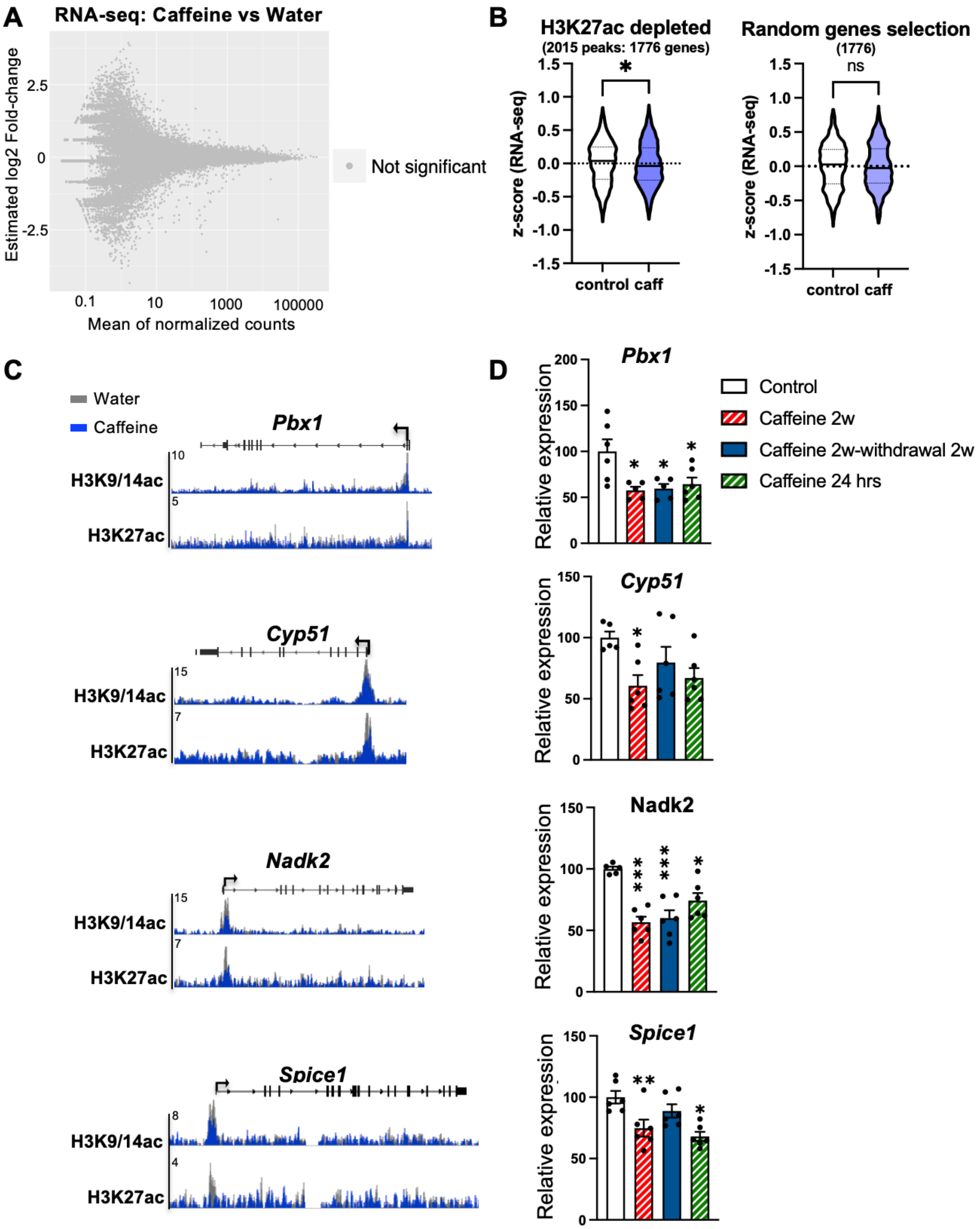
A
RNA-seq: Caffeine vs Water
Estimated log2 Fold-change
Mean of normalized counts
Not significant
B
H3K27ac depleted
(2015 peaks: 1776 genes)
Random genes selection
(1776)
z-score (RNA-seq)
control caff
ns
C
Water
Caffeine
Pbx1
Cyp51
Nadk2
Spice1
H3K9/14ac
H3K27ac
D
Relative expression
Control
Caffeine 2w
Caffeine 2w-withdrawal 2w
Caffeine 24 hrs

Supplemental Figure 2. Hippocampal transcriptomics of water and caffeine-treated mice. (A) MA plot generated from RNA-seq data showing no statistically significant gene expression changes between water and caffeine-treated animals in basal conditions (N=4). (B) Violin plot representation of the integration between RNA-seq and ChIP-seq of H3K27ac depleted genes in caffeine-treated mice showing significant overall decrease in gene expression (z-score) (b) over the same number of randomly chosen genes (test performed in 6 different random datasets). $*p<0.05$ using unpaired two-tailed T-test. (C) Genomic region representation of some metabolic and transcription-related genes found depleted in H3K27ac and H3K9/14ac in caffeine-treated mice. (D) RT-qPCR results of these acetylation depleted genes after acute (24h), chronic treatment (2 weeks) as well as after caffeine withdrawal (2 weeks) (N=5-6). Data are shown as mean ± SEM. $*\ p<0.05$; $**\ p<0.01$; $***p<0.001$ vs control using One-Way ANOVA followed by Tukey's multiple comparison test.

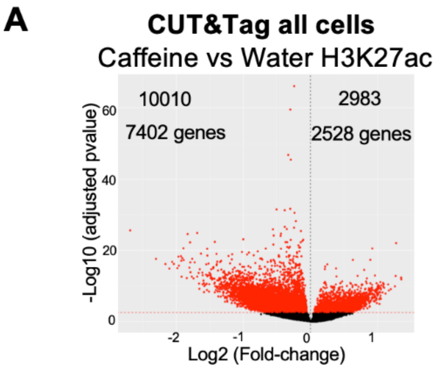

B

H3K27ac depleted

Common upstream regulators	**ChIP** (1945 genes)	CUT&Tag (7402 genes)
TCF7L2	-6.128	-7.932
MKNK1	-4.583	-5.724
NFASC	-2.630	-3.113

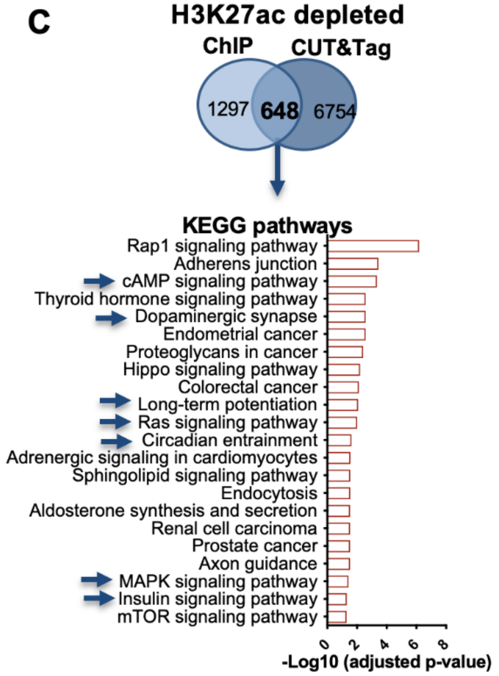

Supplemental Figure 3. H3K27ac CUT&Tag in all cells. (A) Volcano plot showing the differential enriched genomic regions of H3K27ac (CUT&Tag) upon caffeine treatment (10010 decreased and 2983 increased peaks) in all cells. Red dots represent the significant different regions (FDR<0.003). (A) Ingenuity Pathway Analysis (IPA) of ChIP-seq and CUT&Tag decreased H3K27ac genes, showing common upstream regulators in both datasets. (C) Overlap of decreased H3K27ac genes between both epigenetic techniques showing that approximately 30% (648) of the decreased H3K27ac genes by ChIP-seq was found using the CUT&Tag technique. KEGG pathways of the common H3K27ac depleted genes between both techniques. Blue arrows pointing towards the pathways found in H3K27ac ChIP-seq represented in Figure 1e. A total of 2 biological replicates were used for both experiments.

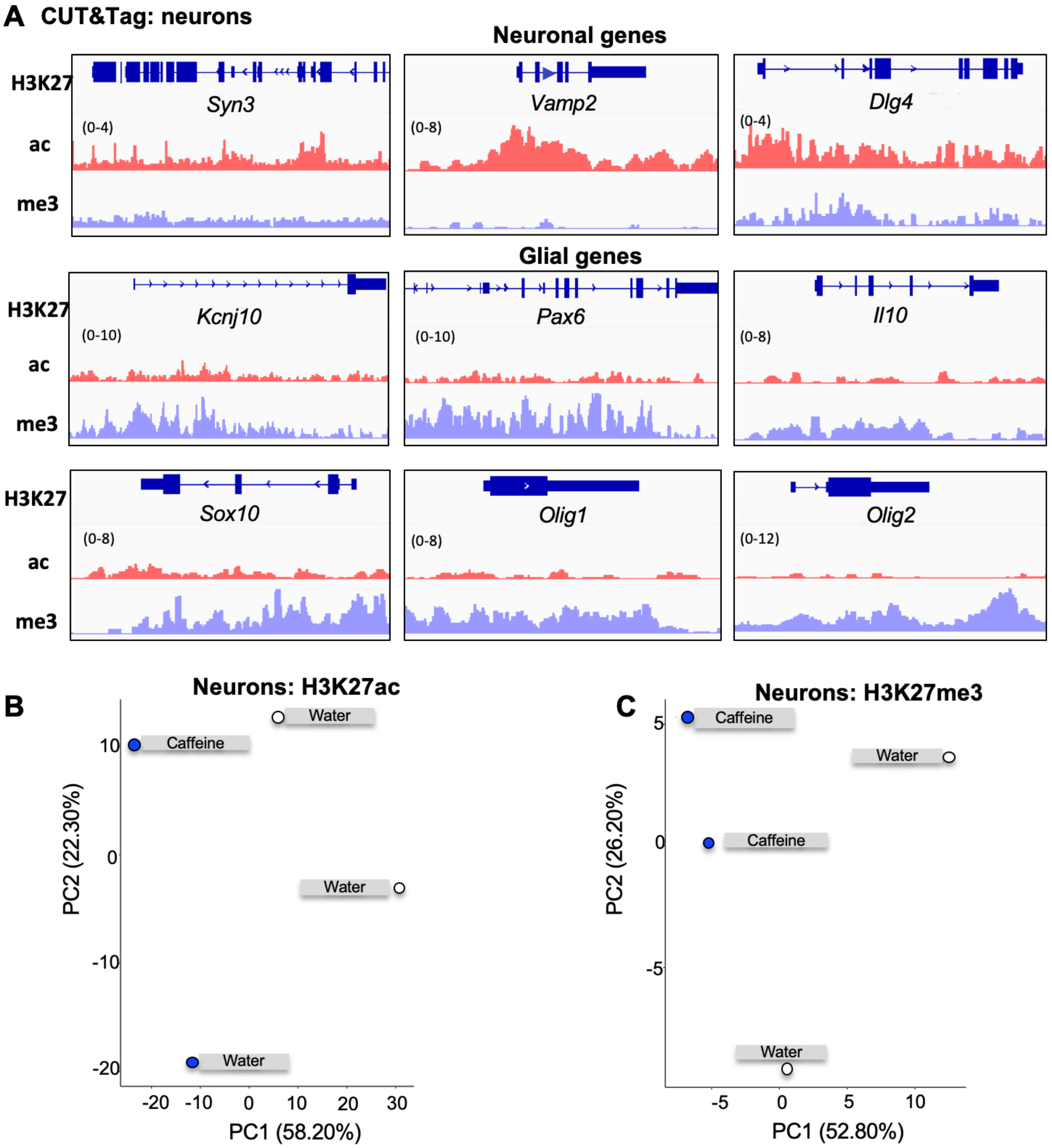

Supplemental Figure 4. Validation of CUT&Tag in neurons. (A) Genomic representation (IGV) of the H3K27ac and H3K27me3 in control samples of neuronal genes, showing higher enrichment in acetylation and depleted in methylation. (B) Genomic representation of glial-related genes demonstrating higher H3K27me3 levels compared with the acetylation ones. (C) PCA plot of control (water-treated) and caffeine-treated mice from CUT&Tag of H3K27ac an (d) and H3K27me3 samples. A total of 2 biological replicates were used per histone mark.

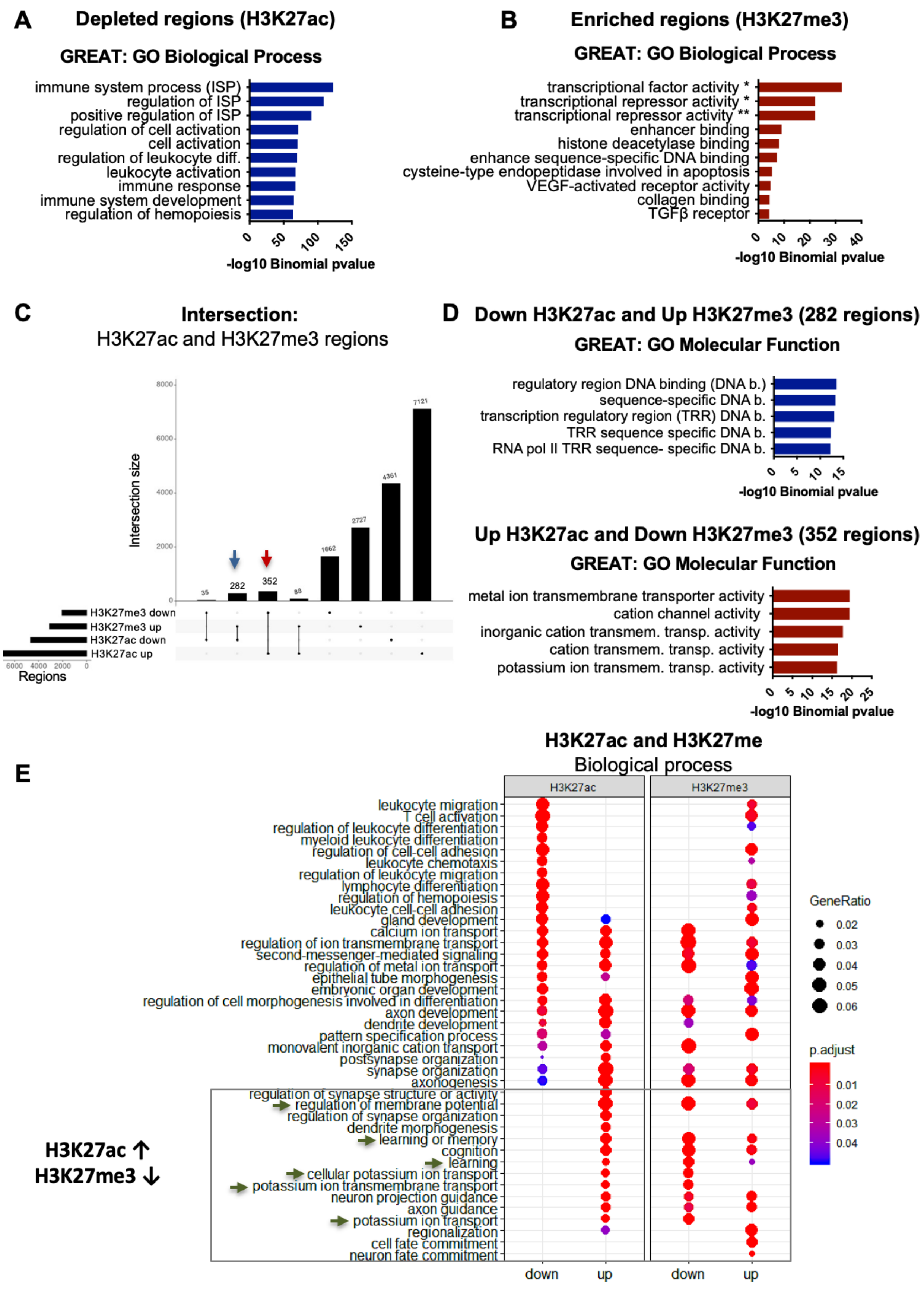
A
Depleted regions (H3K27ac)
GREAT: GO Biological Process
immune system process (ISP)
regulation of ISP
positive regulation of ISP
regulation of cell activation
cell activation
regulation of leukocyte diff.
leukocyte activation
immune response
immune system development
regulation of hemopoiesis
0 50 100 150
-log10 Binomial pvalue
B
Enriched regions (H3K27me3)
GREAT: GO Biological Process
transcriptional factor activity *
transcriptional repressor activity *
transcriptional repressor activity **
enhancer binding
histone deacetylase binding
enhance sequence-specific DNA binding
cysteine-type endopeptidase involved in apoptosis
VEGF-activated receptor activity
collagen binding
TGFβ receptor
0 10 20 30 40
-log10 Binomial pvalue
C
Intersection:
H3K27ac and H3K27me3 regions
Intersection size
8000 6000 4000 2000 0
35 282 352 88 1662 2727 4361 7121
H3K27me3 down
H3K27me3 up
H3K27ac down
H3K27ac up
6000 4000 2000 0
Regions
D
Down H3K27ac and Up H3K27me3 (282 regions)
GREAT: GO Molecular Function
regulatory region DNA binding (DNA b.)
sequence-specific DNA b.
transcription regulatory region (TRR) DNA b.
TRR sequence specific DNA b.
RNA pol II TRR sequence- specific DNA b.
0 5 10 15
-log10 Binomial pvalue
Up H3K27ac and Down H3K27me3 (352 regions)
GREAT: GO Molecular Function
metal ion transmembrane transporter activity
cation channel activity
inorganic cation transmem. transp. activity
cation transmem. transp. activity
potassium ion transmem. transp. activity
0 5 10 15 20 25
-log10 Binomial pvalue
E
H3K27ac and H3K27me
Biological process
H3K27ac
H3K27me3
leukocyte migration
T cell activation
regulation of leukocyte differentiation
myeloid leukocyte differentiation
regulation of cell-cell adhesion
leukocyte chemotaxis
regulation of leukocyte migration
lymphocyte differentiation
regulation of hemopoiesis
leukocyte cell-cell adhesion
gland development
calcium ion transport
regulation of ion transmembrane transport
second-messenger-mediated signaling
regulation of metal ion transport
epithelial tube morphogenesis
embryonic organ development
regulation of cell morphogenesis involved in differentiation
axon development
dendrite development
pattern specification process
monovalent inorganic cation transport
postsynapse organization
synapse organization
axonogenesis
regulation of synapse structure or activity
regulation of membrane potential
regulation of synapse organization
dendrite morphogenesis
learning or memory
cognition
learning
cellular potassium ion transport
potassium ion transmembrane transport
neuron projection guidance
axon guidance
potassium ion transport
regionalization
cell fate commitment
neuron fate commitment
down up down up
H3K27ac ↑
H3K27me3 ↓
GeneRatio
0.02
0.03
0.04
0.05
0.06
p.adjust
0.01
0.02
0.03
0.04

Supplemental Figure 5. Gene Ontology analysis of H3K27ac and H3K27me3 changes induced by caffeine in mouse hippocampal neurons. (A) GREAT analysis of H3K27ac depleted regions and (B) H3K27me3 enriched ones showing the most significant associated Biological Processes. (C) Intersection of depleted/enriched regions between H3K27ac and H3K27me3 (Bedsect V3 tool, overlap size of 100bp, *(Mishra G et al, 2020. BedSect: An Integrated Web Server Application to Perform Intersection, Visualization, and Functional Annotation of Genomic Regions From Multiple Datasets. Frontiers in Genetics, 1664-8021).* (D, blue) GREAT analysis of the regions depleted in H3K27ac and increased in H3K27me3 (282 regions) showing that transcription-related terms are among the most significant molecular functions. (d, red) GREAT analysis of the genomic regions increased in H3K27ac and depleted in H3K27me3 demonstrating that ion transport-related molecular functions are the most significant terms. (E) ClusterProfiler analysis *(Wu T. et al, 202, clusterProfiler 4.0: A universal enrichment tool for interpreting omics data. The Innovation 2(3), 100141)* of depleted/enriched H3K27ac and H3K27me3 showing the most significant deregulated biological processes (adjusted p-value<0.05). Warmer colors represent greater statistical significance and circle size determines the gene ratio within the pathway. H3K27ac enrichment and H3K27me3 depleted genes are related to membrane potential as well as learning and memory processes.
*RNA pol II core promoter proximal region sequence-specific binding
**RNA pol II transcription regulatory region sequence-specific binding

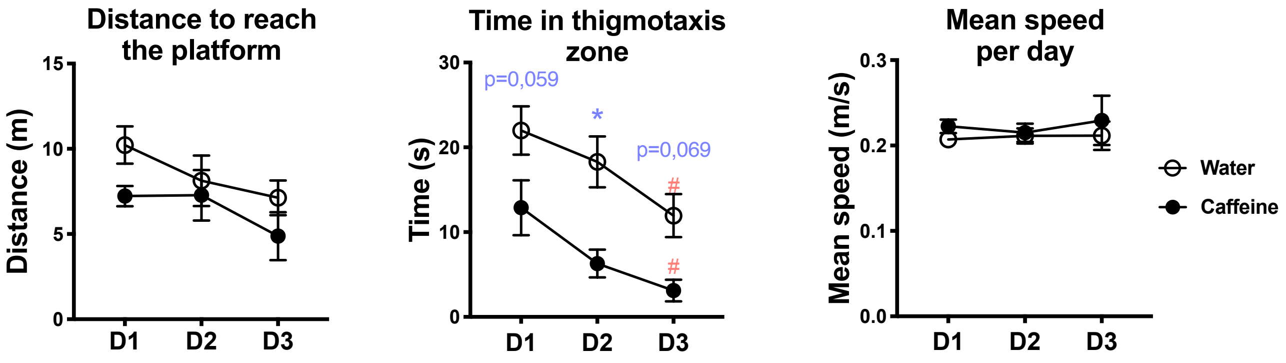

Supplemental Figure 6. Morris-Water maze experiments in water and caffeine-treated animals.
Distance to reach the platform (in meters) during the acquisition period (N=4). Thigmotaxic analysis, represented by the time spent in the periphery of the pool during the 3-days of training, Day 1 and 3 show a tendency for decreased thigmotaxic behavior in the Caffeine group ($p=0.059$ and $p=0.069$, respectively). Swim speed (m/second) during the 3 days for both groups. Data are shown as mean ± SEM. * $p<0.05$ for caffeine vs control using One-Way ANOVA followed by Sidak's multiple comparison test.

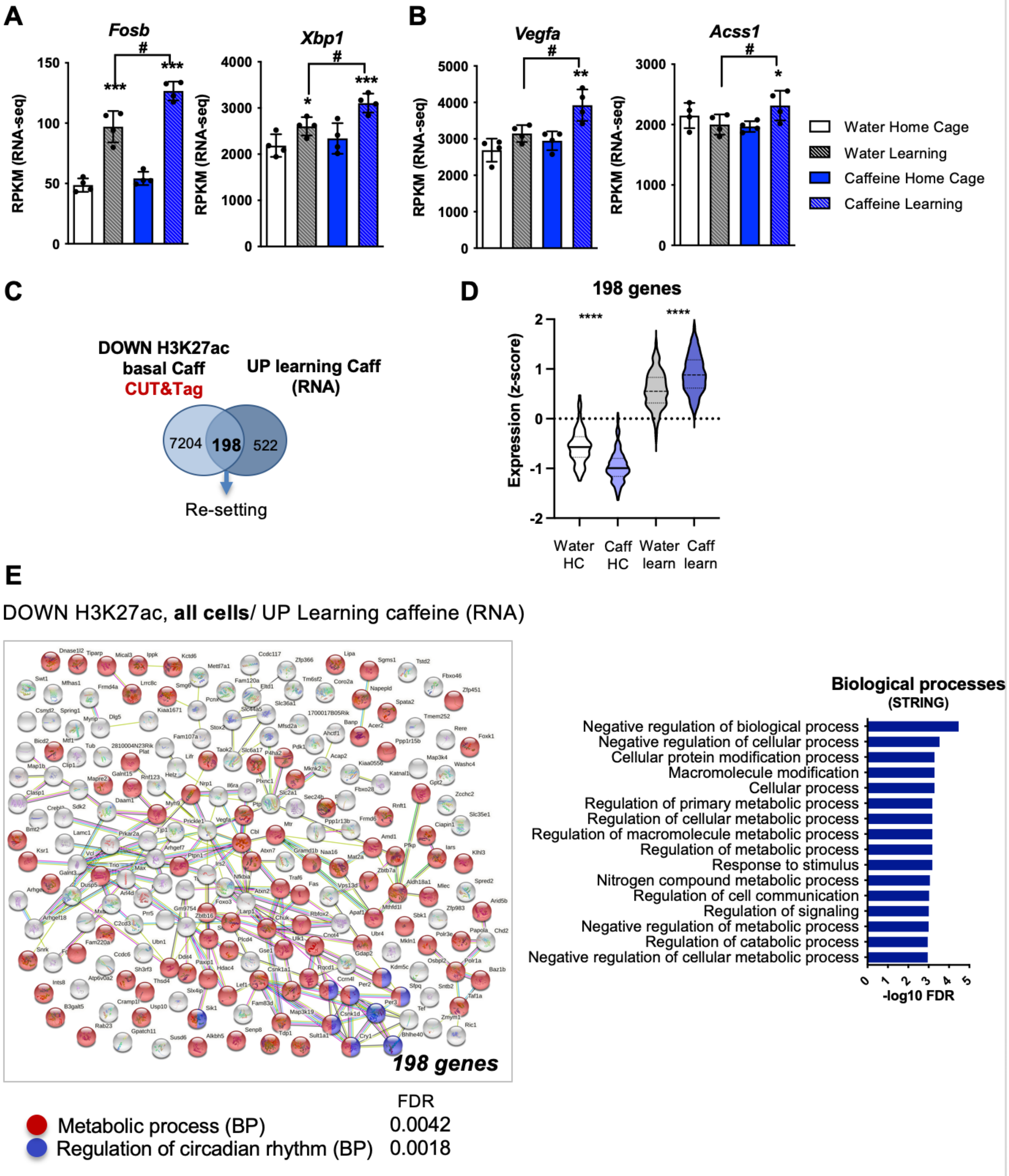
A
Fosb
Xbp1
RPKM (RNA-seq)
B
Vegfa
Acss1
Water Home Cage
Water Learning
Caffeine Home Cage
Caffeine Learning
C
DOWN H3K27ac basal Caff
CUT&Tag
UP learning Caff (RNA)
7204
198
522
Re-setting
D
198 genes
Expression (z-score)
Water HC
Caff HC
Water learn
Caff learn
E
DOWN H3K27ac, all cells/ UP Learning caffeine (RNA)
198 genes
FDR
Metabolic process (BP) 0.0042
Regulation of circadian rhythm (BP) 0.0018
Biological processes (STRING)
Negative regulation of biological process
Negative regulation of cellular process
Cellular protein modification process
Macromolecule modification
Cellular process
Regulation of primary metabolic process
Regulation of cellular metabolic process
Regulation of macromolecule metabolic process
Regulation of metabolic process
Response to stimulus
Nitrogen compound metabolic process
Regulation of cell communication
Regulation of signaling
Negative regulation of metabolic process
Regulation of catabolic process
Negative regulation of cellular metabolic process
-log10 FDR

Supplemental Figure 7. Caffeine-induced transcriptomic alterations upon learning and integration with H3K27ac CUT&Tag dataset (A) RNA-seq data of genes up-regulated by learning in both control and caffeine-treated animals, but significantly more induced in the Caffeine group. (B) Genes up-regulated by learning specifically in the Caffeine group. P-values were retrieved from DESeq2 analysis: * $p < 0.05$; ** $p < 0.01$; *** $p < 0.001$, between learning and home cage conditions. # $p < 0.05$ between both learning groups. (C) Venn diagram showing the overlap of 198 genes that were depleted in H3K27ac (all cells hippocampus CUT&Tag) and up-regulated (RNA-seq) by learning in caffeine-treated mice (re-setting effect). (D) Violin plots of the expression values (z-score) of the 198 genes showing decreased expression in home-cage (HC) and increased in learning conditions between water and caffeine-treated mice. **** $p < 0.0001$ using One-way ANOVA followed Bonferroni's multiple comparison post hoc test. (E) Gene ontology analysis performed with STRING of the 198 genes demonstrating that they are strongly associated with metabolic related biological processes (top 16 by FDR). Data in C-E refers to CUT&Tag all cells and supports the results obtained by ChIP-seq bulk hippocampus (Figure 5 I-K).

Supplemental Table 1. Differential enrichment analysis from H3K9/14ac and ChIP-seq between caffeine treated and control mice hippocampus (xls uploaded).
Supplemental Table 2. Differential enrichment analysis from H3K27ac ChIP-seq between caffeine treated and control mice hippocampus (xls uploaded).
Supplemental Table 3. Canonical pathways (IPA) of epigenomics data (H3K27ac and H3K9/14ac) filtered by Central Nervous System terms (xls uploaded).
Supplemental Table 4. Upstream Regulators (IPA) of epigenomics data (H3K27ac and H3K9/14ac) filtered by Central Nervous System term (xls uploaded).

Most probable assignment based on *m/z* measurements of metabolites and lipids detected in negative and positive ionization modes and differentially expressed in hippocampus from Water and Caffeine animals

Experimental mass (*m/z*)	Precision ΔM expe / theo (ppm)	Intensity Mean ± SEM (a.u.)		*p*-value	Identified species
		Water	Caffeine		
Negative ionization mode					
152.996	1	10659.3 ± 407.2	14039.5 ± 413.0	0.000487	Propanoyl phosphate $[M-H]^-$
	1				Glycerol phosphate $[M-H_2O-H]^-$
524.299	0	17639.4 ± 464.9	22034.3 ± 415.7	0.000200	PS {18:0} $[M-H]^-$
337.092	3	11349.5 ± 435.7	9924.4 ± 190.9	0.038041	p-coumaroylquinic acid $[M-H]^-$
443.279	2	45040.8 ± 3849.4	21942.2 ± 1354.6	0.005798	1,25-dihydroxyvitamin D3-26,23-lactone $[M-H]^-$
447.295	2	14785.1 ± 208.8	13382.9 ± 135.8	0.001815	Vitamin D3 6,19-sulfur dioxide adduct $[M-H]^-$
	3				Cholesterol sulfate $[M-H_2O-H]^-$
827.469	4	24516.0 ± 4405.8	5709.6 ± 1362.7	0.018866	PI {34:5} $[M-H]^-$
828.561	7	20347.7 ± 3804.5	3723.6 ± 1128.7	0.017847	PS {P-42:6} $[M-H_2O-H]^-$
831.539	0	20221.6 ± 3739.0	4659.0 ± 1296.1	0.019488	PI {P-35:2} $[M-H]^-$
842.540	7	10170.0 ± 1108.0	3560.6 ± 541.4	0.004298	PS {42:7} $[M-H_2O-H]^-$
847.577	7	16642.4 ± 1196.0	7821.7 ± 92.2	0.001121	PI {O-36:2} $[M-H]^-$
	7				PI {36:0} $[M-H_2O-H]^-$
855.497	7	22393.2 ± 827.1	11432.1 ± 937.4	0.000025	PI {36:5} $[M-H]^-$
883.532	2	229512.0 ± 11077.1	178182.5 ± 6766.9	0.009912	PI {38:5} $[M-H]^-$
Positive ionization mode					
284.074	0	26332.4 ± 1348.5	30310.3 ± 1121.9	0.047579	Dihydromaleimide beta-D-glucoside $[M+Na]^+$
898.626	4	17646.9 ± 1337.2	32711.7 ± 4679.1	0.022135	PC {43:6} $[M+Na]^+$
170.033	2	83402.3 ± 3374.0	72722.6 ± 2082.5	0.026387	Creatine $[M+K]^+$
270.119	3	105336.6 ± 3957.0	90827.1 ± 1932.4	0.012552	Histidylasparagine $[M+H]^+$
	9				γ-aminobutyryl-lysine $[M+K]^+$
391.287	7	123861.0 ± 1309.7	113458.7 ± 1963.0	0.001838	Bile acid derivate $[M+H]^+$
410.264	7	50019.2 ± 1693.1	41920.0 ± 2059.1	0.013017	Tetradecanoylcarnitine $[M+K]^+$
	7				Myristoylcarnitine $[M+K]^+$
527.075	2	32487.8 ± 1118.4	25189.1 ± 1384.8	0.002347	3-carboxy-1-hydroxypropylthiamine diphosphate $[M+H]^+$
772.454	1	325522.0 ± 3499.6	302339.8 ± 7855.2	0.031209	PS {32:1} $[M+K]^+$
774.508	1	1332720.0 ± 23552.2	1231530.0 ± 35929.2	0.044080	PC {36:8} $[M+H]^+$
	5				PC {34:5} $[M+Na]^+$
844.517	1	1476413.3 ± 33369.5	1363670.0 ± 33724.0	0.038853	Lactosylceramide {d18:1/12:0} $[M+K]^+$
	9				PC {38:6} $[M+K]^+$
848.554	3	3226376.7 ± 89492.7	2947261.7 ± 70569.4	0.035533	PC {38:4} $[M+K]^+$
	9				PI-Cer {d36:0} $[M+Na]^+$

Cer Ceramide; *PC* Phosphocholine; *PI* Phosphoinositol; *PS* Phosphoserine

Supplemental Table 5. Metabolomics data (metabolites and lipids) of control and caffeine-treated mice hippocampus.

Supplemental Table 6. Proteomic alterations of control and caffeine-treated mice hippocampus (xls uploaded).
Supplemental Table 7. Upstream regulators of depleted H3K27ac regions from ChIP-seq and CUT&Tag experiments in bulk hippocampus (xls uploaded).
Supplemental Table 8. H3K27ac and H3K27me3 differential enrichment analysis of caffeine-treated mice in hippocampal neurons (CUT&Tag) (xls uploaded).
Supplemental Table 9. Differential expression analysis (RNA-seq) performed in the hippocampus of caffeine treated and control mice, in basal and upon learning conditions (xls uploaded).
